# Supplementary figures and images for: Malaria parasite centrins can assemble by Ca2+-inducible condensation
Source: PLoS Pathog. 2023 Dec 27;19(12):e1011899. doi: 10.1371/journal.ppat.1011899 (PMC10775985; doi:10.1371/journal.ppat.1011899)

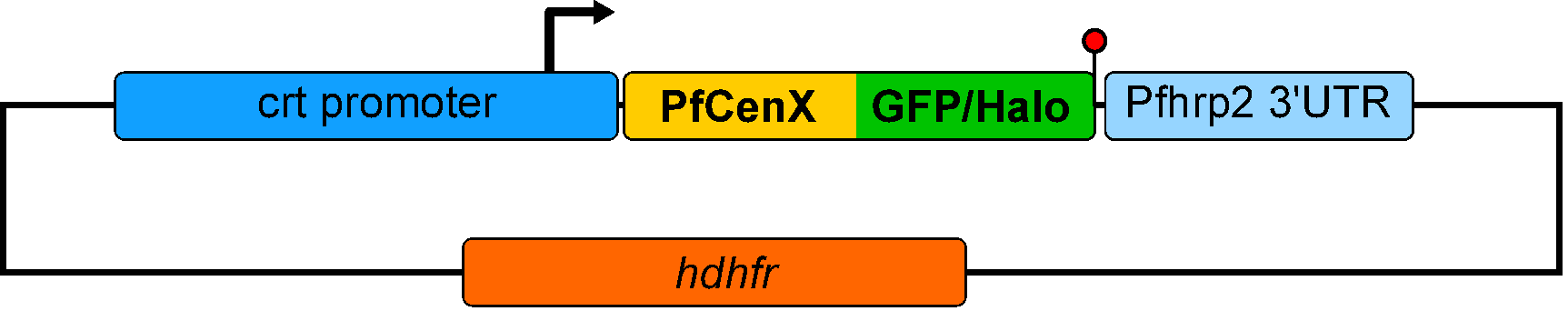

Supplement: S1 Fig — The pARL vector contains a hDHFR cassette, conferring resistance to antifolates, and drives expression of a gene or fusion gene of choice from a weakened promoter of the P. falciparum chloroquine resistance transporter (Pfcrt). (TIF) [file ppat.1011899.s001.tif]

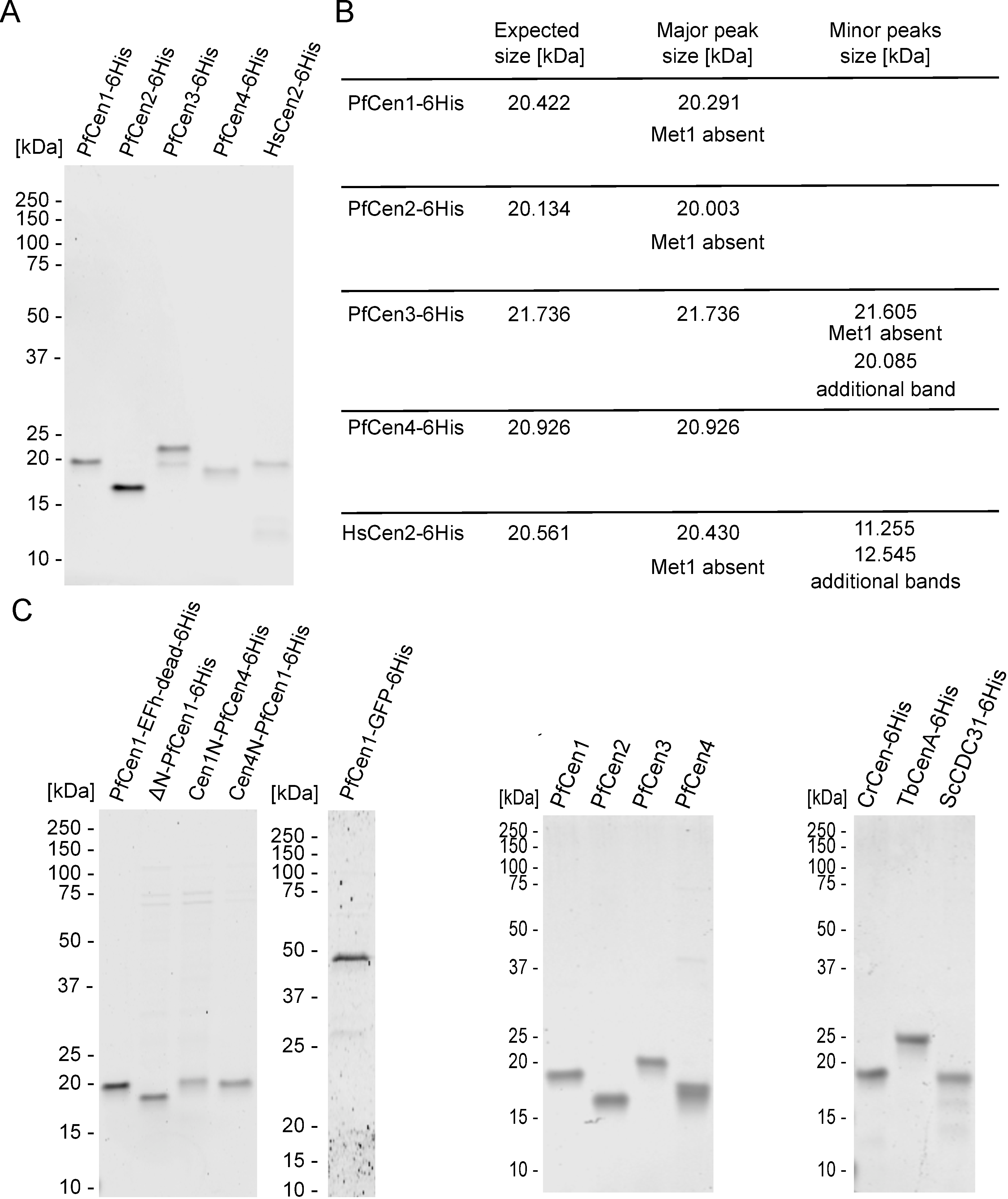

Supplement: S2 Fig — (A) Coomassie gels of wild type centrins recombinantly produced in bacteria and purified via their C-terminal 6His tag show slightly lower migration than expected for PfCen2-6His and PfCen-4-6His (B) Mass spectrometry analysis of native proteins, however, confirms full length expression for all wild type centrins except for cleavage of methionine 1 and occasional detection of degradation bands. (C) Coomassie gels of mutant, tagged and tag-free centrin versions confirms proper expression and show expected migration. Non-plasmodium centrins are T. brucei centrin A (Accession number: XP_846945) 22 kDa, C. reinhardtii centrin (Accession number: P05434) 20 kDa, and S. cerevisiae centrin (Cdc31) 19 kDa. (TIF) [file ppat.1011899.s002.tif]

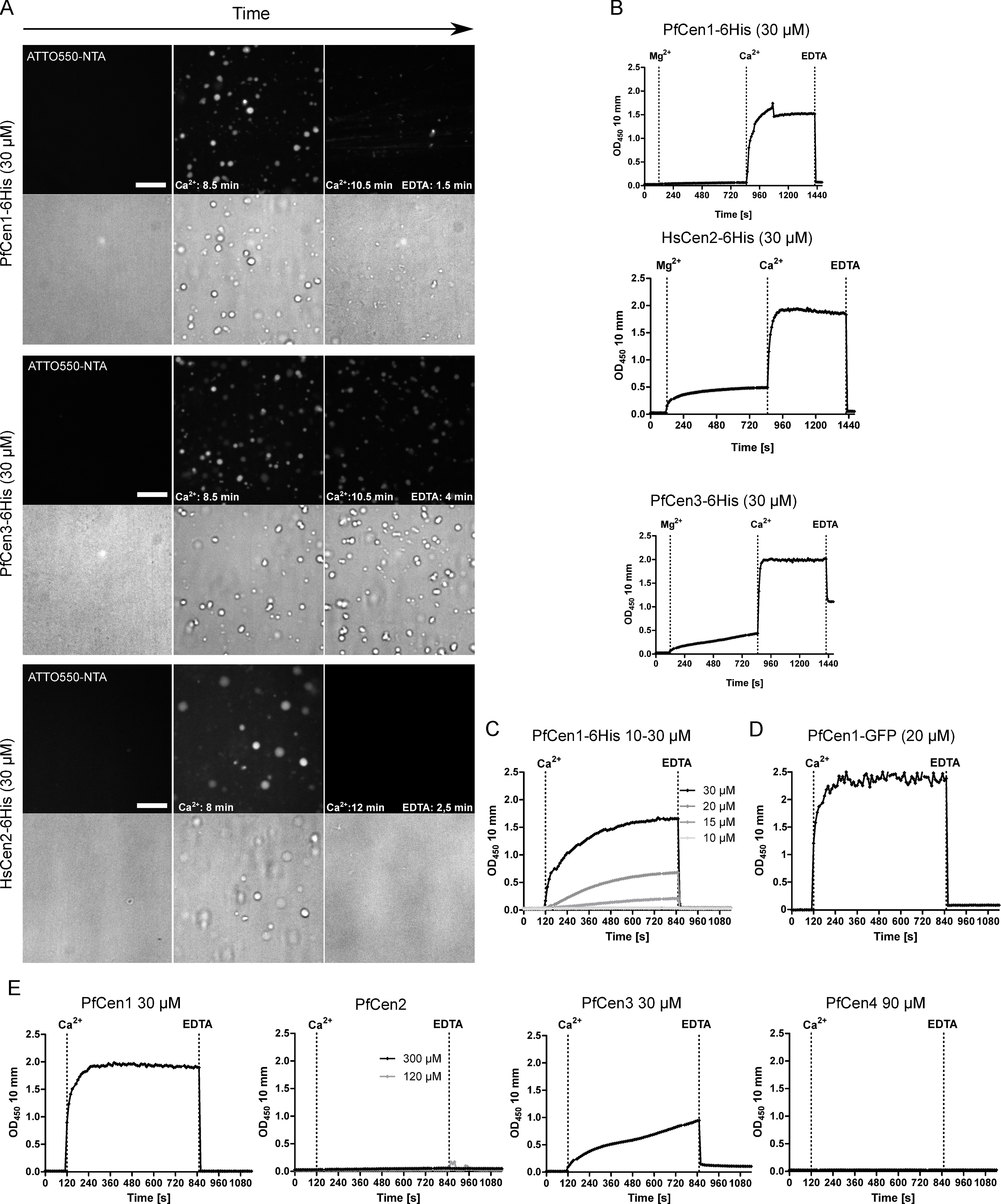

Supplement: S3 Fig — (A) Transmission light images of recombinant PfCen1-6His, PfCen3-6His, and HsCen2-6His centrin protein solutions at 30 μM concentration before, after calcium, and EDTA addition. Proteins were fluorescently labeled via their 6His tag using 300 nM NTA-Atto 550. Time stamps indicate time elapsed between calcium or EDTA addition and image acquisition. Observed droplets are more sparse than in highly concentrated solution but are still dynamic. (B) Turbidity assay using addition of magnesium followed by calcium addition at the same concentration indicates Ca2+ specifically. and not bivalent cations per se, induces centrin LLPS. (C) Testing different PfCen1-6His concentrations reveals saturation concentration to be below 15 μM. (D) Turbidity assay with recombinant PfCen1-GFP at 20 μM. (E) Turbidity assay with recombinant PfCen1-4 after proteolytic cleavage of any protein tag using various concentrations as indicated. Scale bar: 10 μm. Conditions: 50 mM BisTris (pH 7.1), addition of CaCl2 or MgCl2 to 2 mM and EDTA to 10 mM, 37°C. (TIF) [file ppat.1011899.s003.tif]

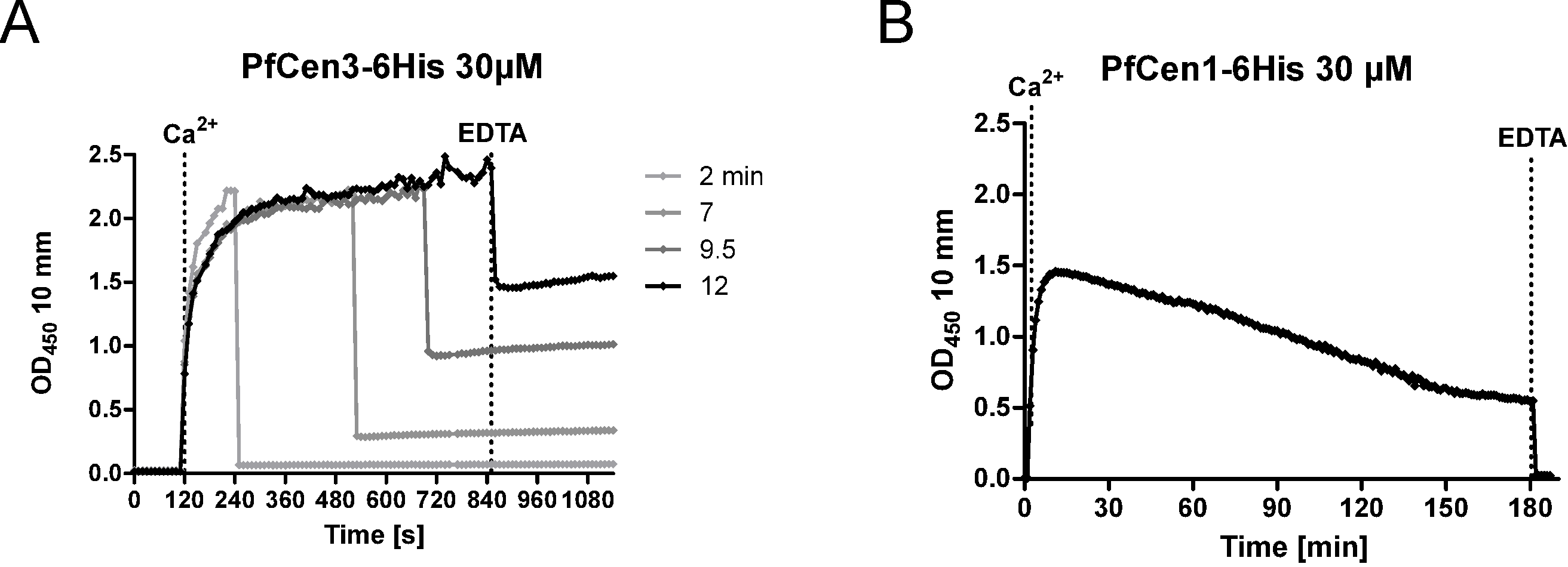

Supplement: S4 Fig — (A) Turbidity of PfCen3-6His solution with varying time points of EDTA addition after Calcium addition. (B) Turbidity of PfCen1-6His protein solution with highly delayed EDTA addition shows no irreversible fraction. Overall reduction of turbidity could be explained by protein droplets settling down. Conditions: 50 mM BisTris (pH 7.1), addition of CaCl2 to 2 mM and EDTA to 10 mM, 37°C. (TIF) [file ppat.1011899.s004.tif]

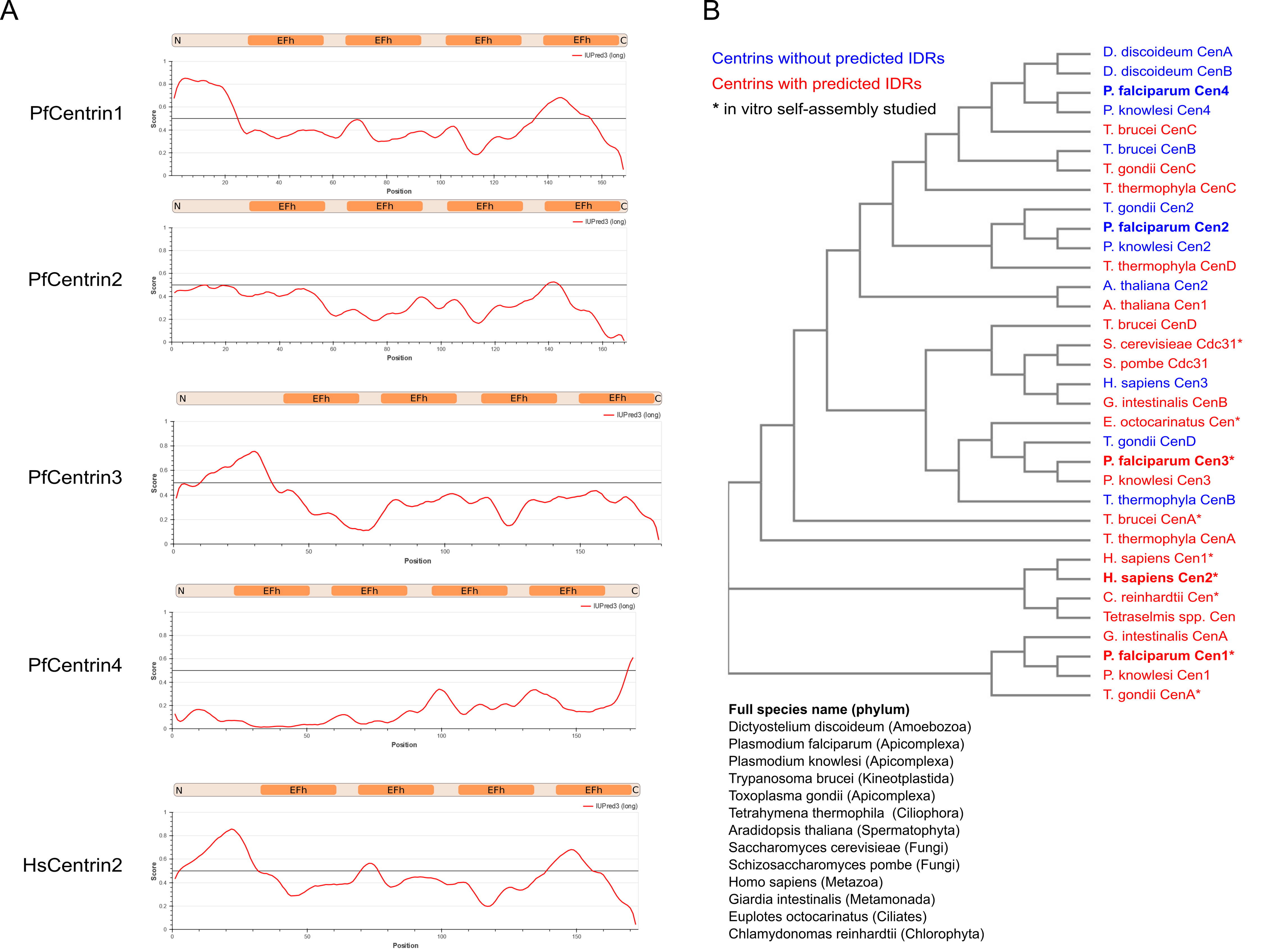

Supplement: S5 Fig — (A) Using the IUpred 3.0 prediction software we identified a higher intrinsic disorder region probability (red line) in centrins undergoing LLPS in vitro. The highest probabilities are found in the N-terminus before the EFh domains. (B) Using Clustal Omega provided by the EMBL European Bioinformatics Institute we created a phylogenetic tree of centrins from multiple highly divergent eukaryotes, for several of which centrin self-assembly has been shown in vitro in this and previous studies (*). Using IUpred 3.0 we determined centrins with an IDR probability above the 0.5 threshold (red) or without increased IDR probability (blue). Centrins analyzed in this study are in bold. (TIF) [file ppat.1011899.s005.tif]

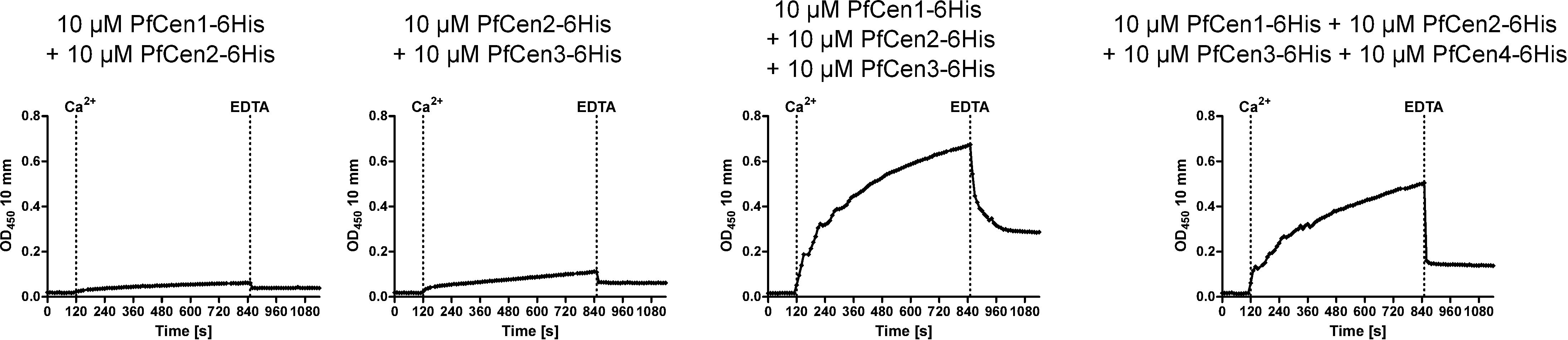

Supplement: S6 Fig — Turbidity measurements of recombinant centrins at individually subcritical concentrations either independently or as a mixture during addition of calcium followed by EDTA as indicated. All conditions: 50 mM BisTris (pH 7.1) at 37°C. (TIF) [file ppat.1011899.s006.tif]

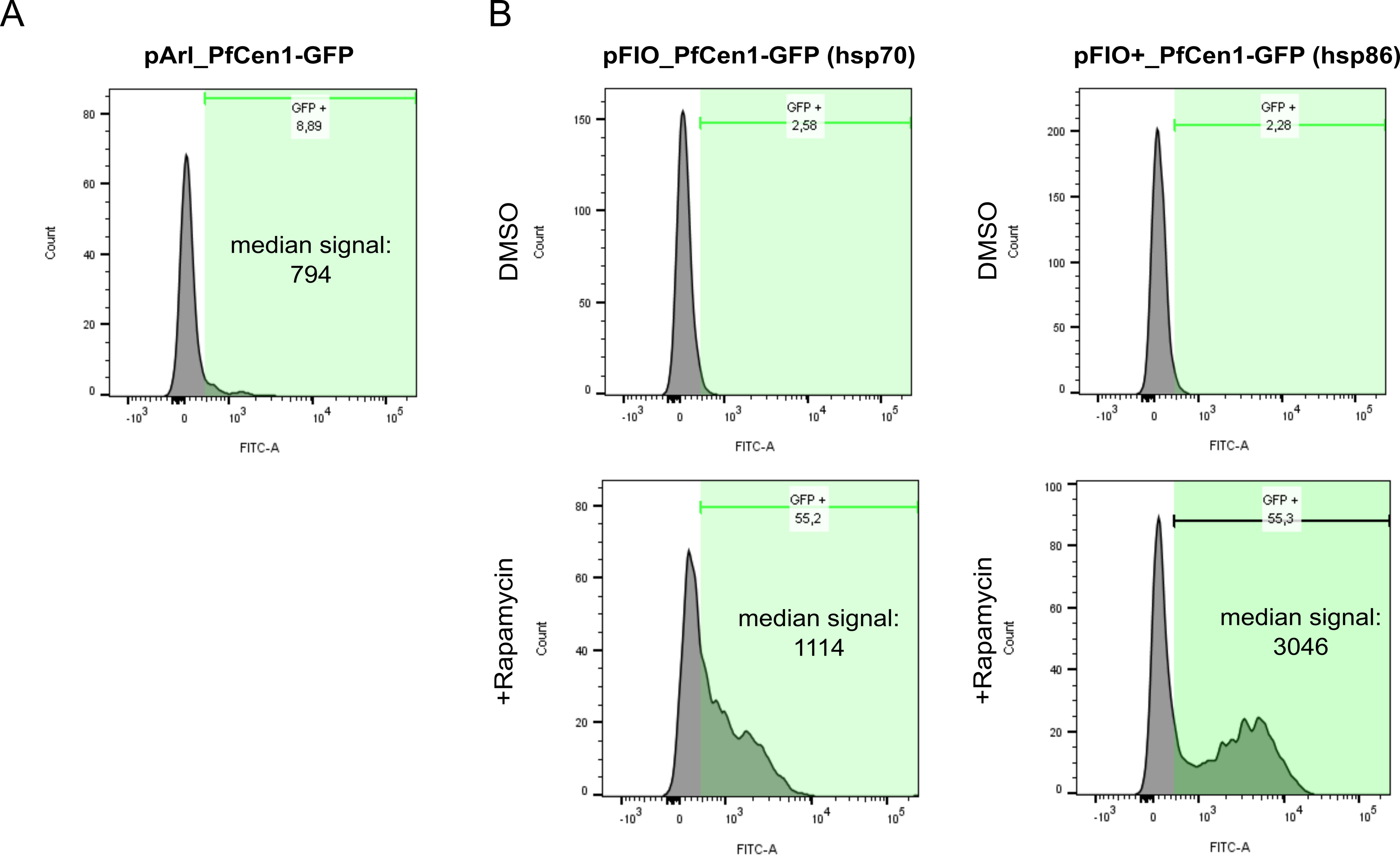

Supplement: S7 Fig — (A) Flow cytometry analysis of multinucleated malaria parasites (stained with SYTO 61 as a DNA marker) expressing PfCen1-GFP using the classical pARL construct only detects a small fraction of GFP-positive cells with a low median fluorescence intensity. (B) Fraction of GFP-positive parasites strongly increases upon induction of pFIO-PfCen1-GFP transfected cells by rapamycin addition. Median GFP fluorescence intensity is much higher than in pARL whereas the hsp86 promoter generates even higher values than the hsp70 promoter fragment. (TIF) [file ppat.1011899.s007.tif]

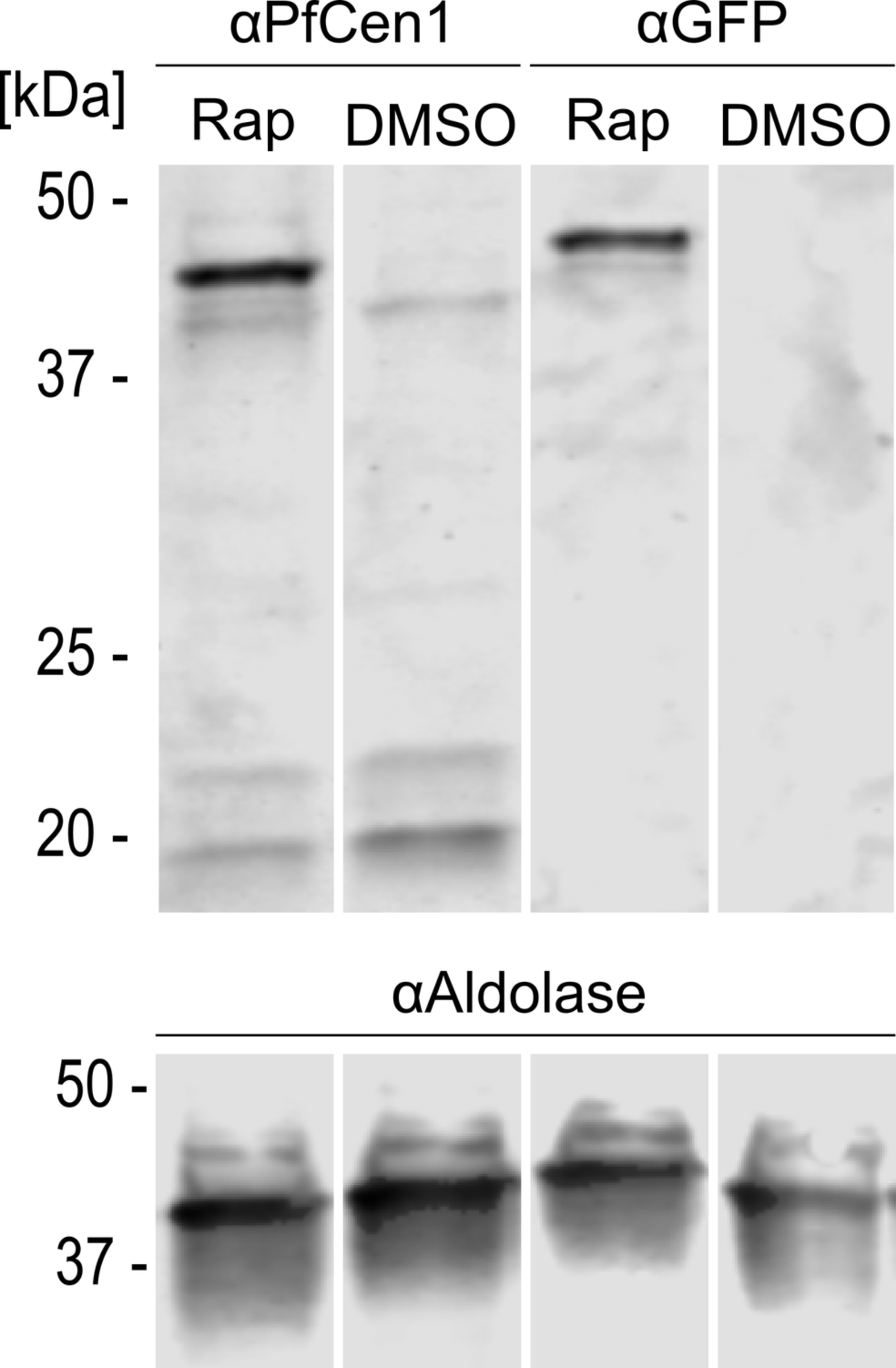

Supplement: S8 Fig — Western blot analysis of purified 3D7-DiCre_pFIO+_PfCen1-GFP late-stage parasites treated with rapamycin or DMSO (control) in the previous cycle. The signal of endogenous centrin (19.6 kDa, 93 AU) and PfCen1-GFP (46.5 kDa, 717 AU) stained with rat anti-PfCen1 was quantified. Corrected for PfCen1-GFP being only present in a subset of the population (43%, n = 662), as quantified via microscopy, an average (induced) cell has an approximate PfCen1:PfCen1-GFP ratio of 1:18. Staining with anti-PfAldolase was employed as a loading control. Anti-GFP staining indicated no cleavage of PfCen1-GFP, with no expression being detected in the DMSO treated populations with either staining. An additional band at ~21 kDa in the anti-PfCen1 staining is likely the result of cross-reactivity with PfCen3 (20.9 kDa). (TIF) [file ppat.1011899.s008.tif]

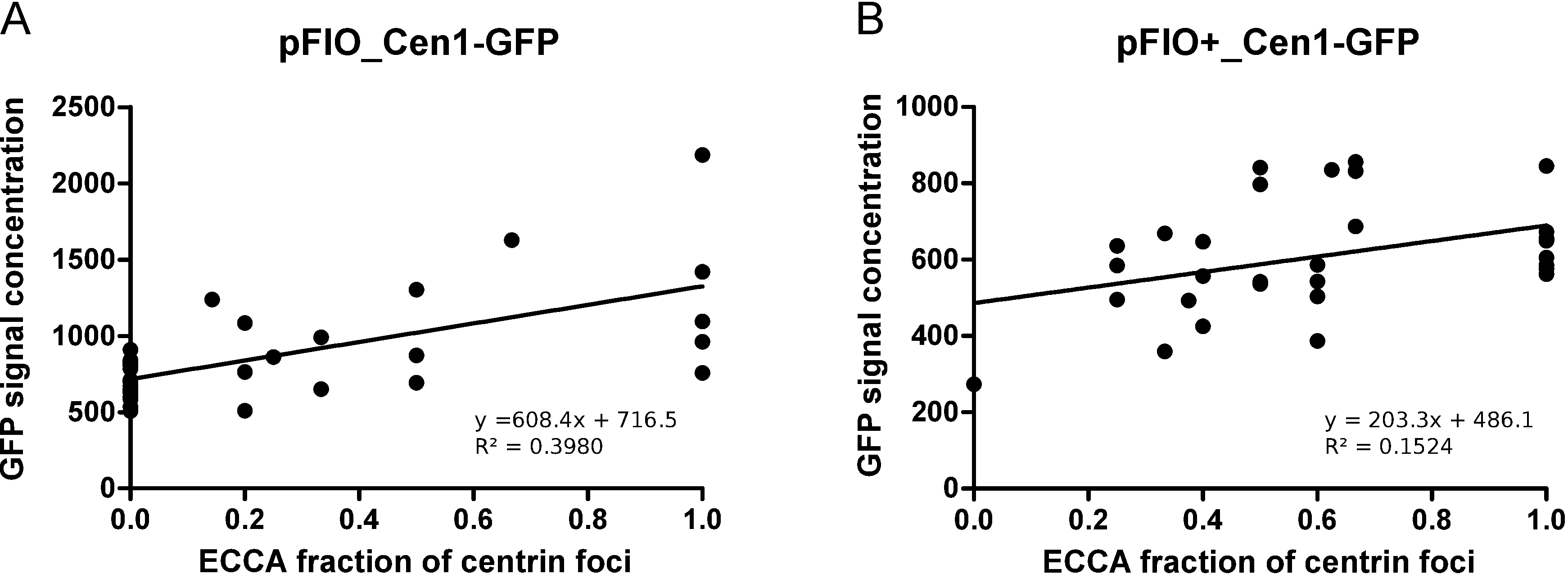

Supplement: S9 Fig — (A) Graph shows total arbitrary GFP fluorescence intensity of cells transfected with pFIO/pFIO+_PfCen1-GFP one asexual cycle after induction, plotted against the fraction of centrin foci, which are ECCAs per centriolar plaque associated centrin foci. Despite the low r-squared value statistical analysis indicate that the slope is significantly not zero (p<0.0001) and therefore positively correlated. (B) as in A but measured for pFIO+_PfCen1-GFP expressing cells. Slope of linear regression is also significantly not zero (p<0.033) as determined by an F-test. Due to the different expression levels different excitation laser settings had to be used for both parasite lines. (TIF) [file ppat.1011899.s009.tif]

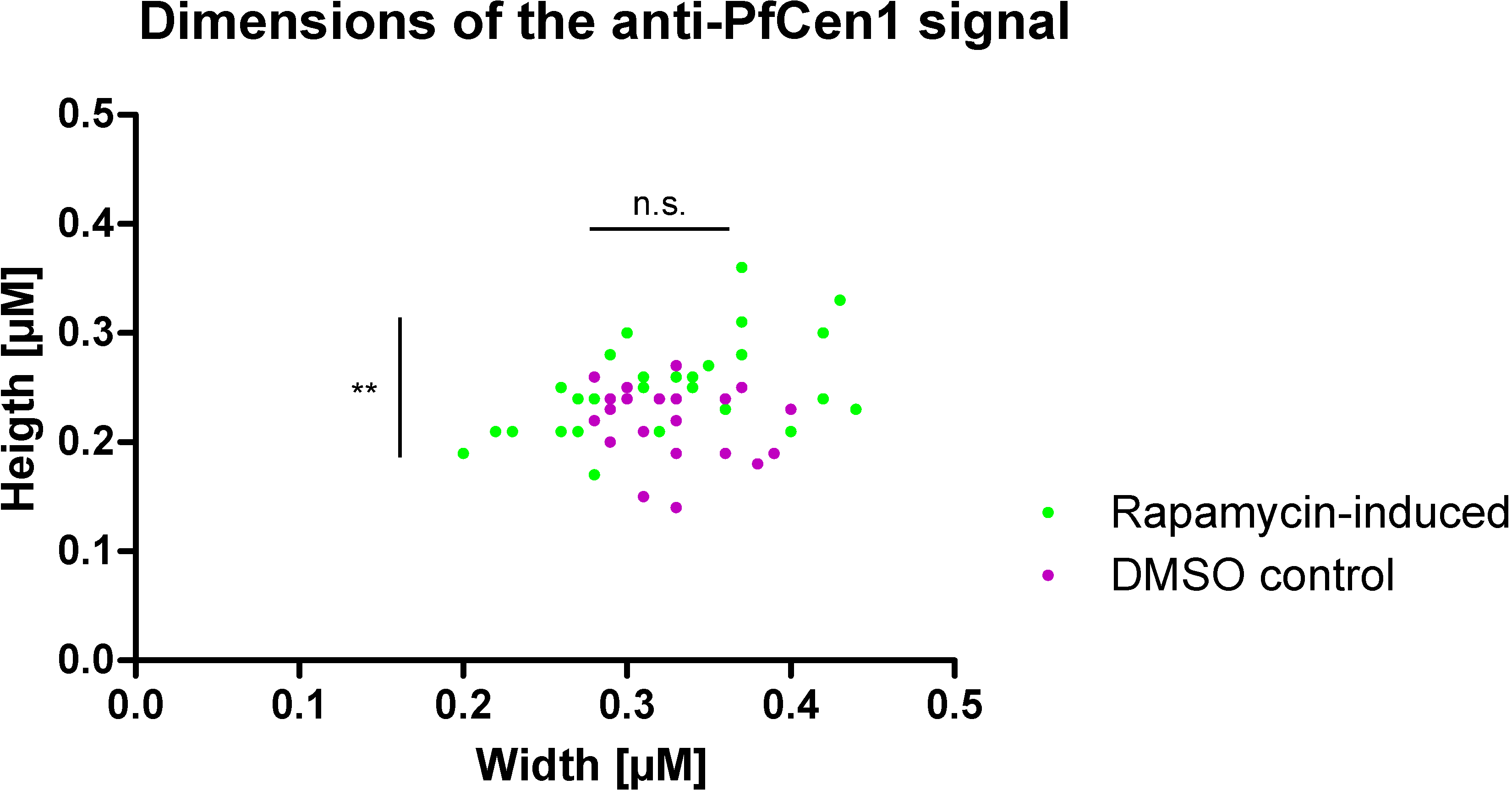

Supplement: S10 Fig — Quantification of immunofluorescence signal at the centriolar plaque measured as in Fig 1D–1E using anti-PfCen1 labeling in rapamycin-induced parasites overexpressing PfCen1-GFP vs control. Using t-test with Welch’s correction width (321 vs 324 nm) shows no difference between the conditions while the height of the signal (247 vs 219 nm) was statistically higher in the induced parasites (p = 0.0081). (TIF) [file ppat.1011899.s010.tif]

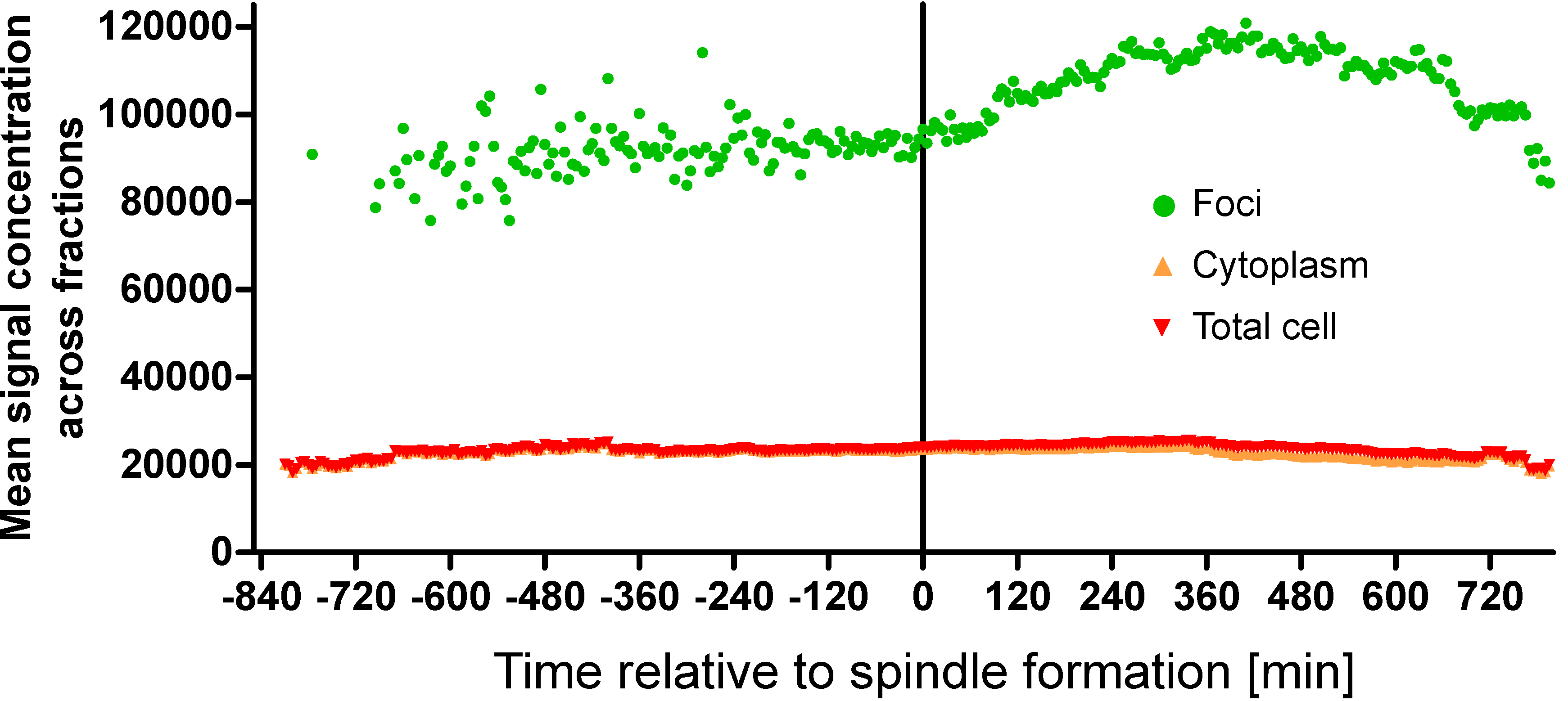

Supplement: S11 Fig — Mean PfCen1-GFP fluorescence intensity by segmented cellular region in induced parasites carrying pFIO+_PfCen1-GFP relative to mitotic spindle formation in time lapse movies. N > 32. (TIF) [file ppat.1011899.s011.tif]

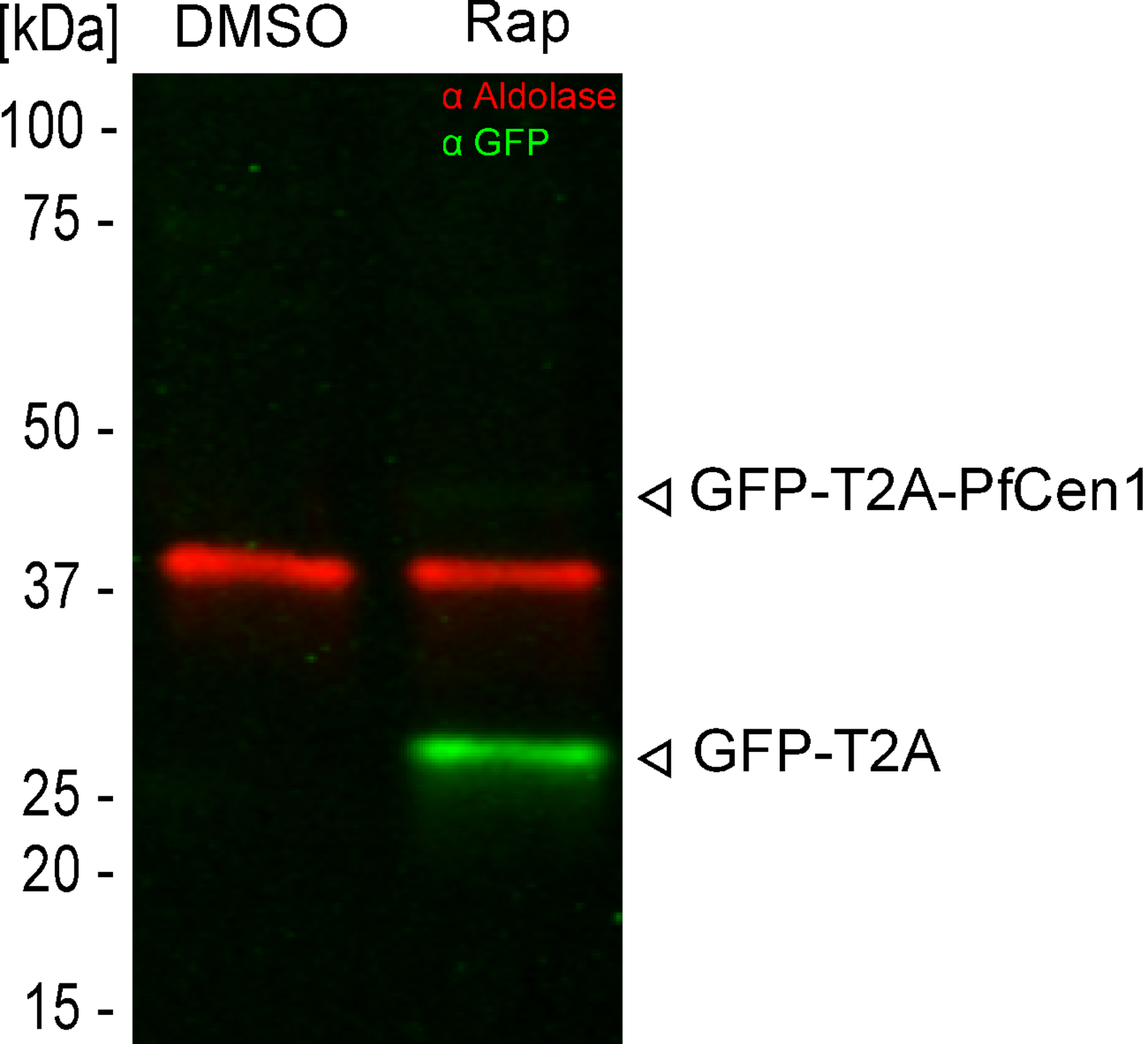

Supplement: S12 Fig — Western blot analysis of protein extract from rapamycin-induced and control late-stage parasites carrying pFIO+_GFP-T2A-PfCen1 plasmid. Anti-GFP antibody was used for detection and anti-PfAldolase as a loading control. Rapamycin-induced parasite extract shows a clear band at the expected size (arrow) for “skipped” GFP while the band for the “unskipped” product is barely detectable. (TIF) [file ppat.1011899.s012.tif]

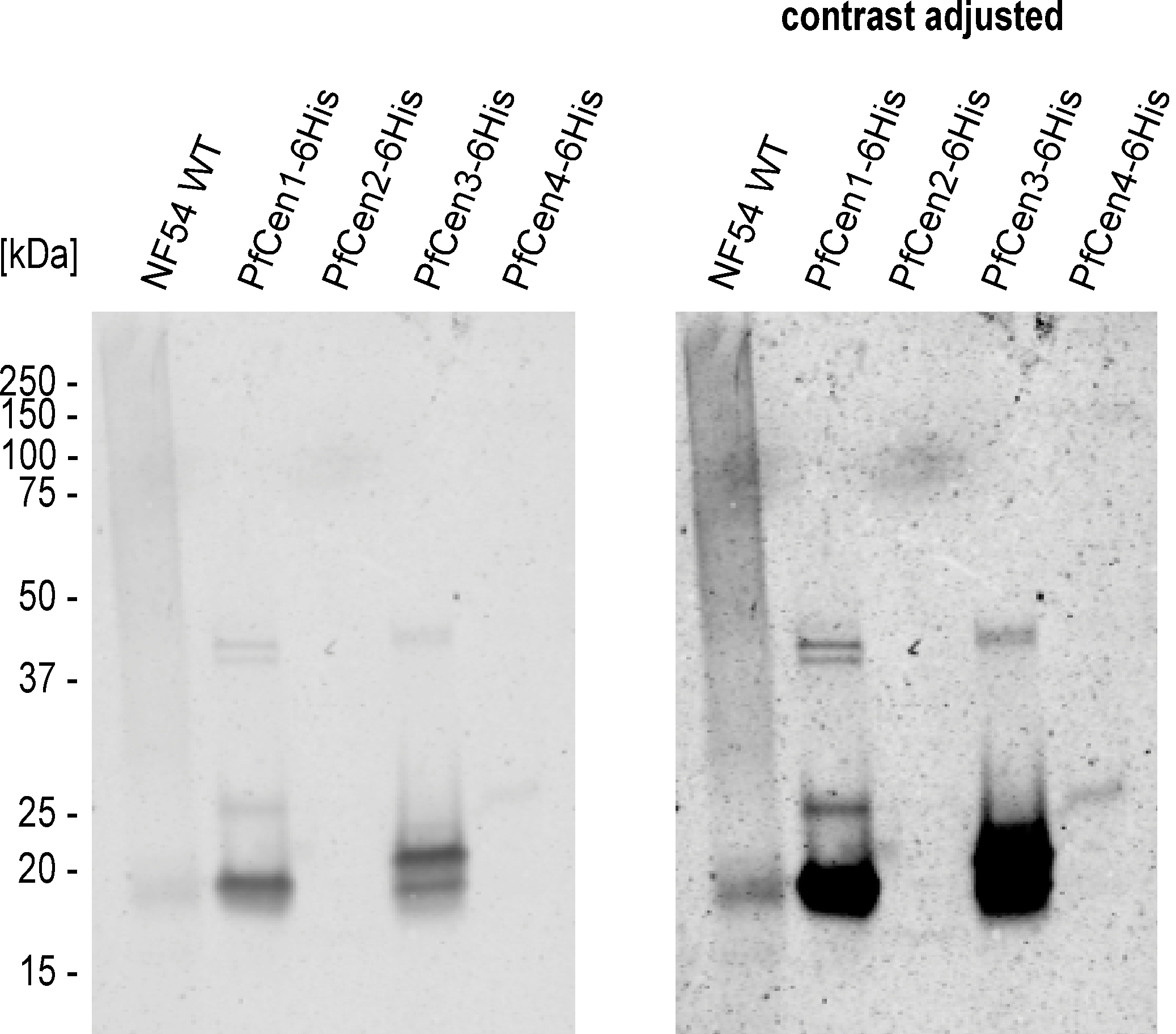

Supplement: S13 Fig — Western blot analysis using newly generated rat anti-PfCen1 antibody. Antibody was tested against total parasite protein extract of late stage wild type NF54 (left lane) and recombinant PfCen1-4-6His proteins (right lanes). Aside some unspecific background the antibody detects a band of the expected size (20 kDa) in parasite extract. Anti-PfCen1 strongly crossreacts with recombinant PfCen3-6His. Two different contrast adjustments of the same blot are shown. (TIF) [file ppat.1011899.s013.tif]

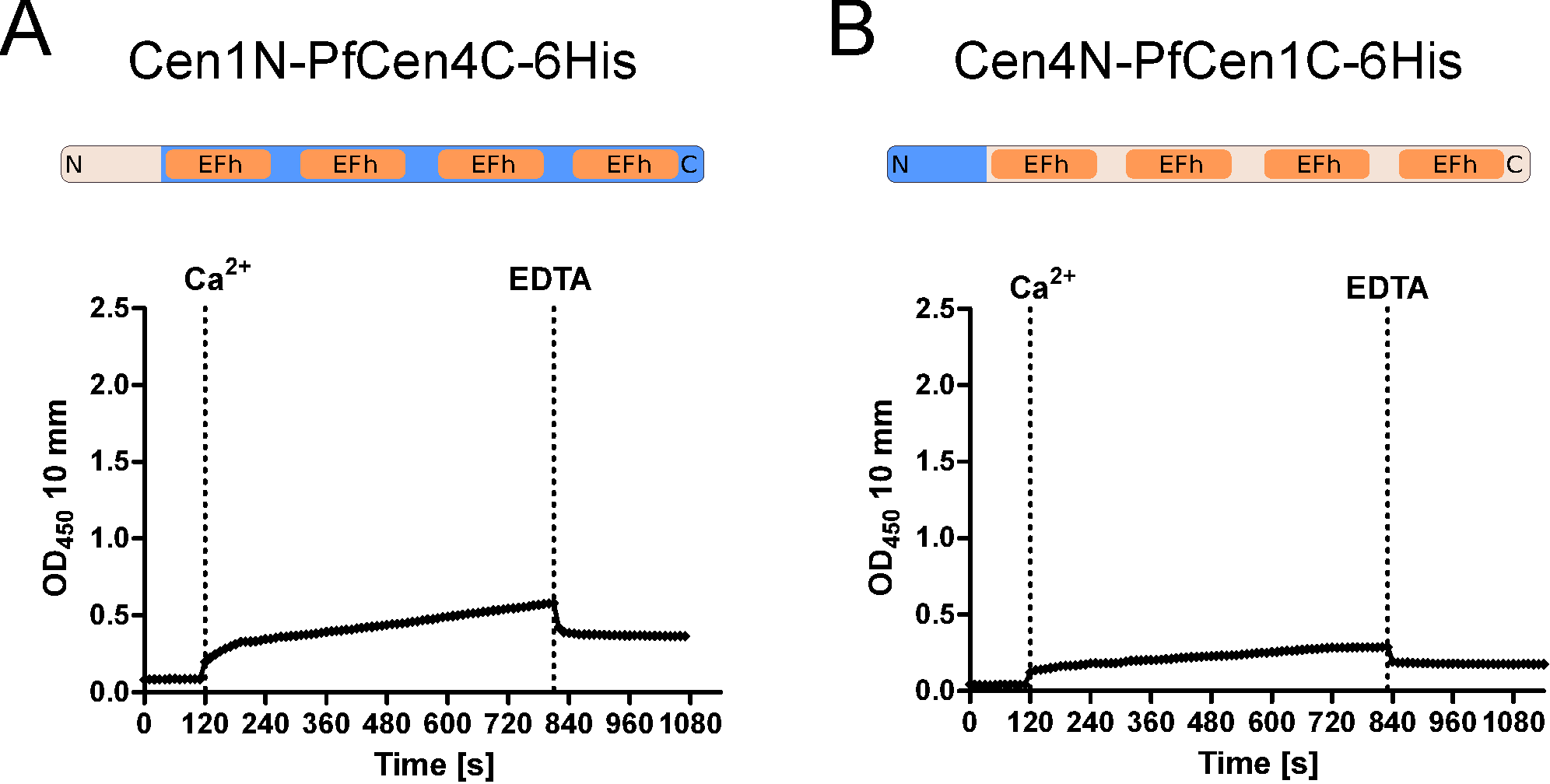

Supplement: S14 Fig — (A) Turbidity of chimeric centrin fused from disordered N-terminus of PfCen1 and EFh domain containing part of PfCen4 during calcium and EDTA addition to protein solution. (B) Turbidity of chimeric centrin fused from N-terminus of PfCen4 and EFh domain containing part of PfCen1 during calcium and EDTA addition to protein solution. Conditions: 50 mM BisTris (pH 7.1), addition of CaCl2 to 2 mM and EDTA to 10 mM, 37°C. (TIF) [file ppat.1011899.s014.tif]

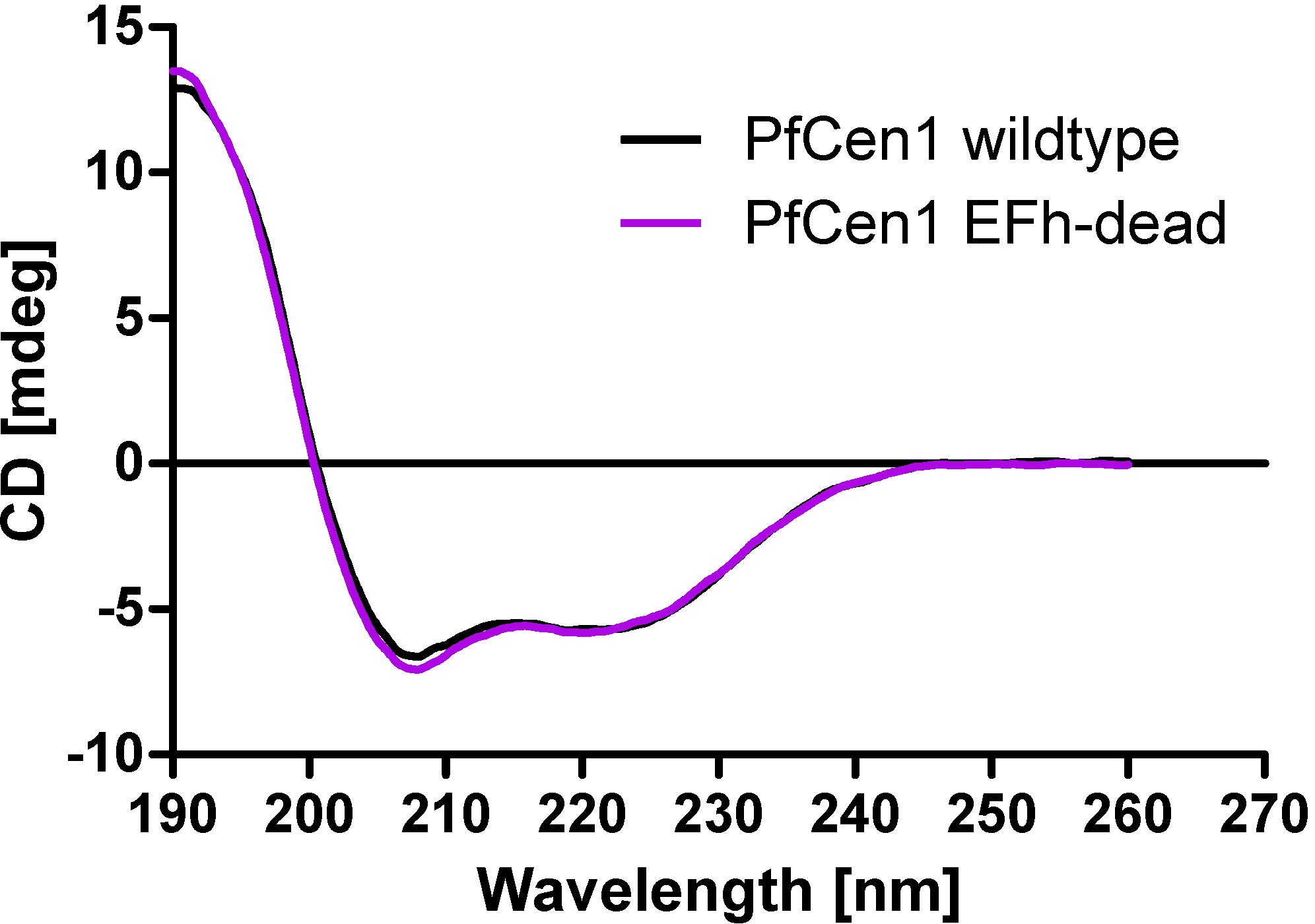

Supplement: S15 Fig — CD spectra of recombinant PfCen1-6His (black) and EFh-dead PfCen1-6His mutant (magenta) protein show no difference. Calculated secondary structure fractions from the CONTIN-LL analysis method of wild type PfCen1 vs mutant PfCen1 yielded 24.7% vs 24.6% helices, 23.0% vs 22.9% strands, 15.8% vs 15.9% turns, and 36.5% vs 36.6% unordered regions, respectively. (TIF) [file ppat.1011899.s015.tif]
