# Supplementary material for: Malaria parasite centrins can assemble by Ca2+-inducible condensation
Source: PLoS Pathog. 2023 Dec 27;19(12):e1011899. doi: 10.1371/journal.ppat.1011899 (PMC10775985; doi:10.1371/journal.ppat.1011899)
Supplement: S1 Table — All primers used for amplification of the various construct fragments used for molecular cloning as described above are listed here. Homology regions for Gibson Assembly are in lowercase, PCR binding sequences are capitalized. * Kindly provided by Dr. Friedrich Frischknecht. (PDF) [file ppat.1011899.s016.pdf]

#	Amplicon	Template	Dir.	Sequence
P1	PfCen1	NF54 cDNA	F	cgacccgggatggtaccATGAGCAGAAAAAATCAAACTATG
P2	PfCen1	NF54 cDNA	R	ttcttctcctttactcctaggAAATAAGTTGGTCTTTTTCATAATTC
P3	PfCen2	NF54 cDNA	F	cgacccgggatggtaccATGACCGATAACACAGCTG
P4	PfCen2	NF54 cDNA	R	ttcttctcctttactcctaggTAAGAAGCTTTTTTTGGTCATTATTG
P5	PfCen4	NF54 cDNA	F	cgacccgggatggtaccATGAACACAATGTTAATTAAGG
P6	PfCen4	NF54 cDNA	R	ttcttctcctttactcctaggTGAATCTGAATCAACATCAC
P7	Halo+Linker	p238-actin-chromobody-halo*	F	gaaaaagaccaacttatttcctaggCCAACCACTGAGGATCTGT
P8	Halo+Linker	p238-actin-chromobody-halo*	R	ccctcgaggaattcctgcaggtctggacatTTAACCGGAAATCTCCAGAGTAGAC
P9	PfHsp70 5' UTR	NF54 gDNA	F	cactatagaatactcgcggccgcCGCATAAATATCTGGTGAAATACAAAC
P10	PfHsp70 5' UTR	NF54 gDNA	R	atgtatgctatacgaagttatgaaCCTTTTGCACTAGCCAATTTTTC
P11	hDHFR+GGGS linker	pARL-Cen3-GFP	F	cttcgtatagcatacattatacgaagttatATGCATGGTTCGCTAAAC
P12	hDHFR+GGGS linker	pARL-Cen3-GFP	R	aatctattattaaataaatggatccacctccaccATCATTCTTCTCATATACTTC
P13	hDHFR+GGGS linker	pARL-Cen3-GFP	R	TCTTCTCCCTTAGATACGG
P14	mCherry	p3xNLS-mCherry-hsp86-BSD	F	gaatgatggtggaggtggatccGTATCTAAGGGAGAAGAG
P15	mCherry	p3xNLS-mCherry-hsp86-BSD	R	gtataatgtatgctatacgaagttatgtcgacttaTGTAGAGTGTCTACCTTC
P16	GFP	pARL-Cen3-GFP	F	gtatagcatacattatacgaagttatacgcgtATGGTTAGTAAAGGAGAAGAACTTTTC
P17	GFP	pARL-Cen3-GFP	R	ctattattaaataaatgctcgagTTATTTGTATAGTTCATCCATG
P18	PbDHFR 3'UTR	PbANKA gDNA	F	gtagacactctacataagtcgacGATATGGCAGCTTAATGTTC
P19	PbDHFR 3'UTR	PbANKA gDNA	R	atgctatacgaagttat G ATATCGAAATTGAAGG
P20	PfHsp86 5'UTR	NF54 gDNA	F	cactatagaatactcgcggccgcGGGAATTTCCTTATAAGATCTTTC
P21	PfHsp86 5'UTR	NF54 gDNA	R	atgtatgctatacgaagttATTTTATTCGAAATGTGGGAAG
P22	Cen1	pARL-Cen1-GFP	F	acattatacgaagttatacgcgtATGAGCAGAAAAAATCAAAC
P23	ΔN-PfCen1	pARL-Cen1-GFP	F	acattatacgaagttatacgcgtATGGAATTAAATGAAGAACAAAAATTAG
P24	GFP-T2A	pSLI-TGD_PfSlp-GFP [42]	F	acattatacgaagttatacgcgtATGAGTAAAGGAGAAGAACTTTTC
P25	GFP-T2A	pSLI-TGD_PfSlp-GFP [42]	R	agtttgatttttctgctTGGTCCTGGATTTTCTTCTAC
P26	PfCen1	pFIO+_PfCen1-GFP	F	AGCAGAAAAAATCAAACTATG
P27	PfCen1	pFIO+_PfCen1-GFP	R	Ctattattaaataaatgctcgag TTA AAATAAGTTGGTCTTTTTCATAATTC
P28	HsCen2	human cDNA	F	actttaagaaggagatataccATGGCCTCCAACTTTAAGAAGG
P29	HsCen2	human cDNA	R	agtggtggtggtggtggtgcccATAGAGGCTGGTCTTTTTCATG
P30	PfCen1 (E. coli)	oligo 6	F	actttaagaaggagatataccATGTCTCGTAAAAACCAAACC
P31	PfCen1 (E. coli)	oligo 6	R	agtggtggtggtggtggtgcccGAATAAGTTAGTTTTTTCATAATGCG

P32	ΔN-PfCen1 (E. coli)	oligo 6	F	tttaagaaggagatataccatgGAGCTGAATGAGGAACAAAAG
P33	PfCen1 (E. coli)	oligo 6	R	ttctcctttactcctaggGAATAAGTTAGTTTTTTTCATAATGCG
P34	PfCen2 (E. coli)	oligo 7	F	actttaagaaggagatataccATGACAGATAACACTGCC
P35	PfCen2 (E. coli)	oligo 7	R	agtggtggtggtggtggtgcccCAGGAAACTTTTCTTGGTC
P36	GFP	pARL-Cen3-GFP	F	CCTAGGAGTAAAGGAGAAGAAC
P37	GFP	pARL-Cen3-GFP	R	agtggtggtggtggtggtgcccTTTGTATAGTTCATCCATGCC
P38	PfCen3 (E. coli)	oligo 8	F	ttaaagaggagaaaggatccATGATTAACCGTAAGAGC
P39	PfCen3 (E. coli)	oligo 8	R	gtggtgatgatggtgATATAAGGATGTCTGCTTC
P40	PfCen4 (E. coli)	oligo 9	F	ttaaagaggagaaaggatccATGAATACTATGCTGATCAAAG
P41	PfCen4 (E. coli)	oligo 9	R	gtggtgatgatggtgAGAGTCGCTGTCAACGTC
P42	PfCen1 (E. coli)	oligo 6	F	aacctgtattttcagggcATGTCTCGTAAAAACCAAACC
P43	PfCen2 (E. coli)	oligo 7	F	aacctgtattttcagggcATGACAGATAACACTGCC
P44	PfCen3 (E. coli)	oligo 8	F	aacctgtattttcagggcATGATTAACCGTAAGAGCG
P45	PfCen4 (E. coli)	oligo 9	F	aacctgtattttcagggcATGAATACTATGCTGATCAAAG
P46	pZE13d ORF		R	CAGCTAATTAAGCTTGGCTGCAG

S1 Table. Primers used in this study. All primers used for amplification of the various construct fragments used for molecular cloning as described above are listed here. Homology regions for Gibson Assembly are in lowercase, PCR binding sequences are capitalized. * Kindly provided by Dr. Friedrich Frischknecht.
