# Supplementary material for: Malaria parasite centrins can assemble by Ca2+-inducible condensation
Source: PLoS Pathog. 2023 Dec 27;19(12):e1011899. doi: 10.1371/journal.ppat.1011899 (PMC10775985; doi:10.1371/journal.ppat.1011899)
Supplement: S2 Table — Lowercase letters indicate Gibson assembly or ligation overhangs. All listed ds DNA oligos used for molecular cloning were ordered as GeneArt Strings (ThermoFisher). (PDF) [file ppat.1011899.s017.pdf]

Oligos	Encodes	ss/ds	Sequence
oligo 1	EFh-dead PfCen1 aspartate 37, 73, 110, 146 mutated to alanine	dsDNA	acattatacgaagttatacgcgtATGAGCAGAAAAAATCAAACTATGAT<br>AAGGAACCCTAATCCTCGAAGTAAAAGAAACGAATTAAAT<br>GAAGAACAAAAATTAGAAATTAAGGAAGCTTTTGATTTAT<br>TTGCTACAAACGGCACTGGAAGAATTGATGCAAAAGAATT<br>AAAAGTTGCAATGAGAGCTTTAGGATTCGAACCAAAAAAA<br>GAAGATATAAGGAAAATAATATCTGATGTTGCTAAAGATG<br>GATCTGGTACAATTGACTTTAATGACTTTTTAGACATTATG<br>ACAATAAAAATGAGTGAAAGAGATCCTAAGGAGGAAATA<br>CTAAAAGCTTTCCGATTATTTGCTGATGATGAGACTGGAAA<br>AATCTCCTTCAAAAATTTAAAACGTGTAGCAAAAGAACTT<br>GGAGAGAATATTACTGATGAGGAAATTCAAGAAATGATAG<br>ACGAAGCAGCCAGAGATGGAGACGGAGAAATTAATGAAG<br>AAGAATTTATGAGAATTATGAAAAAGACCAACTTATTTccta<br>ggagtaaaggagaagaa
oligo 2	HsCen2<br><br>codon optimized for *P. falciparum*	dsDNA	acattatacgaagttatacgcgtATGGCAAGTAATTTTAAAAAAGCAAAT<br>ATGGCAAGTTCAAGTCAAAGAAAAAGAATGAGTCCAAAAC<br>CAGAATTAACAGAAGAACAAAAACAAGAAATTAGAGAAgc<br>aTTTGATTTAttcGATGCAGATGGAACTGGAACAATAGATGT<br>AAAAGAATTAAAAGTAGCAATGAGAGCATTAGGATTTGAA<br>CCTAAAAAAGAAGAAATTAAAAAAATGATAAGTGAAATA<br>GATAAAGAAGGAACTGGAAAAATGAATTTTGGAGATTTTctt<br>ACAGTAATGACACAAAAAATGAGTGAAAAAGATACAAAA<br>GAAGAAATATTAAAAGCTTTTAAACTTTTTGATGATGATGA<br>AACTGGAAAAATTtccTTTAAAAATTTAAAAAGAGTAGCTA<br>AAGAActtGGAGAAAATTTAACAGATGAAGAATTACAAGAA<br>ATGATAGATGAAGCAGATAGAGATGGAGATGGAGAAGTAT<br>CAGAACAAGAATTTTTAAGAATTATGAAAAAAACATCATT<br>ATATcctaggagtaaaggagaagaa
oligo 3	Stop codon	ssDNA	gaagaatgatggtggaggtggatccTAAgtcgacgatatggcagcttaatgtt
oligo 4	6His	ssDNA	gatcCATACGCGTCGACACCATCATCACCACCACTAACtgca
oligo 5	6His	ssDNA	GTTAGTGGTGGTGATGATGGTGTCGACGCGTATG
oligo 6	PfCen1<br><br>codon optimized for *E. coli*	dsDNA	catcaccaccacatcgatATGTCTCGTAAAAACCAAACCATGATTCGT<br>AATCCGAATCCGCGTAGCAAACGCAACGAGCTGAATGAGG<br>AACAAAAGCTTGAAATTAAAGAAGCGTTCGATCTTTTTGAC<br>ACAAATGGTACAGGGCGTATCGACGCAAAAGAGCTTAAAG<br>TGGCAATGCGCGCATTGGGGTTTGAACCAAAAAAGGAGGA<br>CATCCGTAAAATTATTTCAGATGTCGATAAAGACGGCTCTG<br>GGACGATTGATTTCAATGACTTTTTAGACATTATGACCATT<br>AAAATGTCGGAACGCGATCCCAAAGAGGAAATTCTTAAAG<br>CGTTTCGTTTATTCGACGACGACGAAACCGGAAAAATTTCC<br>TTCAAAAACCTGAAGCGCGTAGCAAAAGAGCTTGGAGAAA<br>ATATCACGGACGAGGAAATTCAAGAGATGATTGATGAAGC<br>GGACCGCGATGGAGATGGAGAAATTAACGAGGAAGAGTTC<br>ATGCGCATTATGAAAAAAACTAACTTATTCTAActgcagccaagc<br>ttaatt
oligo 7	PfCen2<br><br>codon optimized for *E. coli*	dsDNA	catcaccaccacatcgatATGACAGATAACACTGCCGTGCGCCGCCG<br>TTATGAAAAATCTTTCACGGAACGTCCGGGTTTTACGGAGG<br>ATGAGATTGAGGAAATTCGTGAAGCCTTTAACCTTTTGGAC<br>ACCGACGGGACGGGGACCATCGATCCTAAGGAAATTAAAT<br>GTGCCATGCAATCGCTTGGCTTAGATGCAAAGAATCCCATG<br>ATTTTCCGCATGATCGCGGACCTGGAAAAAGACGGCTATTC<br>CTCCATCGATTTTGAAGAATTCATGGAGGTGATTACAAGTA<br>AGTTGGGCAACAAGGACACGCGTGAGGGCATTCAGCGCAT<br>CTTCAACTTATTTGATGACGATAAAACAGGTTCGATCTCTC<br>TTAAAAACTTAAAACGTGTTGCTAAAGAGTTAGGCGAAAC<br>ATTAACGGACGAAGAATTGCGTGATATGATTGATCGTGCC<br>GATTCGAAGGGGGAGGGCGAAATCAGCTTCGAGGACTTTT<br>ACACTATTATGACCAAGAAAAGTTTCCTGTAActgcagccaagctt<br>aatt

oligo 8	PfCen3<br><br>codon optimized for *E. coli*	dsDNA	catcaccaccacatcgatATGATTAACCGTAAGAGCGAATCTGTCAG<br>CTACACGCCTCGTGCAATCACTAACCGCCCTATTAGTAGCA<br>ATCGCCGTCGCGGGCGCAACGAAATTACAGACGAGCAGAA<br>AAATGAAATTAAGGAAGCGTTTGATCTTTTCGATACAGAG<br>AAAACCGGCAAAATCGACTATCATGAGCTGAAGGTTGCCA<br>TCCGTGCATTAGGATTCGATATTAAGAAAGCGGACGTTTTA<br>GACTTGATGCGCGAATACGACAAGACCAACAGTGGGCATA<br>TCGATTATAACGATTTTCTTGACATTATGACTCAAAAGATT<br>TCTGAGCGTGACCCAACGGAAGAAATTATTAAGGCTTTTA<br>AGCTTTTCGATGACGACGATACTGGGAAAATCTCCTTGAAG<br>AATTTGCGTCGTGTTTCGCGTGAGCTGGGTGAAAATTTAAG<br>CGATGACGAGTTACAAGCTATGATCGACGAATTCGACAAA<br>GACATGGATGGGGAAATCTCTCAAGAGGAATTCTTGTCAA<br>TCATGAAGCAGACATCCTTATATTAActgcagccaagcttaatt
oligo 9	PfCen4<br><br>codon optimized for *E. coli*	dsDNA	catcaccaccacatcgatATGAATACTATGCTGATCAAAGATAACAT<br>TAACATCTCAATTAACGAGGATGTAGAAAAAGAACTGTAC<br>GAATGCTTTTCACTGTTTGACACGAACAAGTGCGGCTACAT<br>TGATATTCGTGAGTTTTACTTTGCCTTGAAATCCTTGGGCCT<br>GAACTTCAAGAAGGAGCAGGTTAAAAATATTTTTTTGGAC<br>ATCAAGAAAGATATTGACGAGAAATTGAATTTTGACGAAT<br>TTTTTGATATCGCAACGAAATATCTGCATACCCGCTATAAT<br>GACGACGAAATGGACCAAATGTTCTCATTGTTTGACCCCAA<br>CGATACTGGTAAAATTACACTTCAGGCTCTGCGTAAGGTTT<br>GCACTGACATCGGGGAGAATATCTCGGATACCGAGCTGAA<br>TAACATGATCCACTTCGCAGACAAAAACAATGATAAAGTC<br>ATCGATAAAAACGAGTTCAAAAAGGTGCTTCTTTGCTCCTG<br>GAAGAATGATCCGTTATCGGACGTTGACAGCGACTCTTAAc<br>tgcagccaagcttaatt
oligo 10	EFh-dead PfCen1 aspartate 37, 73, 110, 146 mutated to alanine. Codon optimized for E. coli	dsDNA	actttaagaaggagatataccATGTCTCGTAAAAACCAAACCATGATTC<br>GTAATCCGAATCCGCGTAGCAAACGCAACGAGCTGAATGA<br>GGAACAAAAGCTTGAAATTAAAGAAGCGTTCGATCTTTTT<br>GCCACAAATGGTACAGGGCGTATCGACGCAAAAGAGCTTA<br>AAGTGGCAATGCGCGCATTGGGGTTTGAACCAAAAAAGGA<br>GGACATCCGTAAAATTATTTCAGATGTCGCGAAAGACGGC<br>TCTGGGACGATTGATTTCAATGACTTTTTAGACATTATGAC<br>CATTAAAATGTCGGAACGCGATCCCAAAGAGGAAATTCTT<br>AAAGCGTTTCGTTTATTCGCCGACGACGAAACCGGAAAAA<br>TTTCCTTCAAAAACCTGAAGCGCGTAGCAAAAGAGCTTGG<br>AGAAAATATCACGGACGAGGAAATTCAAGAGATGATTGAT<br>GAAGCGGCCCGCGATGGAGATGGAGAAATTAACGAGGAA<br>GAGTTCATGCGCATTATGAAAAAAACTAACTTATTCgggcacc<br>accaccaccaccact
oligo 11	ScCDC31<br><br>codon optimized for *E. coli*	dsDNA	actttaagaaggagatataccATG TCC AAA AAT CGC TCC AGT TTG<br>CAG TCA GGA CCG TTG AAC TCG GAG TTG TTG GAG GAA<br>CAA AAG CAG GAG ATT TAC GAG GCA TTT TCA CTG TTT<br>GAT ATG AAT AAT GAC GGT TTT TTA GAC TAC CAT GAG<br>TTA AAA GTC GCT ATG AAA GCA CTG GGA TTC GAA TTA<br>CCC AAA CGT GAA ATT CTT GAT TTG ATT GAC GAG TAT<br>GAC TCG GAG GGT CGC CAT TTA ATG AAA TAT GAT GAT<br>TTT TAC ATC GTA ATG GGC GAA AAA ATT CTG AAA CGT<br>GAC CCG CTG GAC GAG ATC AAG CGT GCG TTC CAG TTG<br>TTT GAT GAC GAT CAT ACA GGC AAG ATC AGC ATT AAA<br>AAC TTA CGT CGC GTG GCA AAG GAA CTG GGT GAG ACC<br>CTG ACG GAT GAG GAG TTG CGC GCA ATG ATC GAG GAG<br>TTT GAC CTT GAT GGC GAT GGG GAG ATC AAT GAG AAC<br>GAG TTC ATC GCA ATC TGT ACT GAT AGT<br>TTCgggcaccaccaccaccaccact
oligo 12	TbCen2	dsDNA	actttaagaaggagatataccATG TCT ACT ACC AAC CGT GGA GGT<br>AAT TCG CCA GTG GCC CAG AGT GTG AAC CGT AGC ATC<br>GCT CCG GGC ATG TCA CTT AGT GGC TCC GCC CTG GCG<br>GTA CTT ACT GAG GAA CAA CGT CAG GAA ATC AAA GAA

	codon optimized for *E. coli*		GCA TTT GAC CTT TTT GAT ACA GAT GGA TCT GGG ACC ATC GAT GTC AAA GAA TTG AAA GTT GCG ATG CGC GCC TTG GGG TTC GAG CCC CGT AAG GAC GAG GTA CGT CGT CTG ATC GCT TCC ACG ATG GAG GAA CGC GGA GAT CCC GCT CCA GTG AAA CCT GGC ACC GCG CCG GGA GCC GAC AAC CAT GTC ATC GGG TTC GCC GAG TTC GTC GAT TTG ATG GCC CGT AAA ATG AAC GAA CGT GAT TCC CGT GAA GAG ATG TTG AAA GCT TTC CAC TTA TTT GAT GAC GAT AAA ACA GGT AAA ATT ACT TTT AAA AAC TTA AAG CGT GTG GCT CAG GAA TTA GGT GAA AAC ATG ACC GAT AGT GAA ATT CAA GAG ATG ATC GAT GAA GCA GAC CGC GAC GGA GAT GGA GAG GTC AGT GAG GAA GAG TTC CTG CGT ATT ATG AAG AAG ACG AGT CTT TAT TTCgggcaccaccaccaccaccact
oligo 13	CrCen<br><br>codon optimized for *E. coli*	dsDNA	actttaagaaggagatatacc ATG TCA TAC AAG GCA AAA ACC GTA GTT TCG GCC CGC CGC GAC CAA AAG AAA GGA CGC GTC GGT CTT ACC GAG GAG CAG AAA CAA GAA ATC CGC GAA GCG TTT GAT TTA TTC GAC ACT GAT GGA AGT GGG ACC ATC GAC GCC AAG GAA TTA AAG GTG GCG ATG CGC GCT TTA GGA TTC GAA CCC AAG AAG GAA GAG ATC AAA AAA ATG ATT TCG GAA ATT GAT AAG GAC GGC TCC GGC ACA ATC GAC TTT GAG GAA TTT TTA ACG ATG ATG ACA GCG AAG ATG GGT GAA CGT GAT AGC CGT GAG GAA ATT CTT AAA GCA TTC CGT CTG TTC GAC GAT GAC AAC AGT GGC ACC ATT ACG ATC AAG GAC TTA CGC CGT GTG GCA AAG GAG TTG GGG GAG AAT TTA ACC GAA GAG GAG TTG CAA GAA ATG ATT GCC GAA GCC GAT CGT AAT GAT GAT AAT GAA ATT GAT GAG GAC GAA TTT ATT CGT ATT ATG AAA AAG ACG AGT CTG TTCgggcaccaccaccaccaccact
oligo 14	6His-Linker-TEV	ssDNA	atcatcaccaccacatcgatTACGATATCCCAACGACCGAAAACCTGTATTTTCAGGGCGCCACGCGTctgcagccaagcttaattagctgat

S2 Table. Oligos used in this study. Lowercase letters indicate Gibson assembly or ligation overhangs. All listed ds DNA oligos used for molecular cloning were ordered from Genestring.
