# Supplementary material for: Malaria parasite centrins can assemble by Ca2+-inducible condensation
Source: PLoS Pathog. 2023 Dec 27;19(12):e1011899. doi: 10.1371/journal.ppat.1011899 (PMC10775985; doi:10.1371/journal.ppat.1011899)
Supplement: S3 Table — List of antibodies with information of species, final dilution and source with order number. Starred (*) are dilutions for confocal IFAs. For STED microscopy concentration was increased to 1:200. (PDF) [file ppat.1011899.s018.pdf]

| **Antibody** | **Species** | **Dilution** | **Source** |
|---|---|---|---|
| Anti-alpha-tubulin B-5-1-2, monoclonal | mouse | 1:500 | Sigma-Aldrich (T5168) |
| Anti-PfCentrin3, polyclonal | rabbit | 1:500 | Simon et al. 2021 |
| Anti-PfCentrin1, polyclonal | rat | 1:500<br>WB<br>1:1000 | This study |
| Anti-PfAldolase | Rabbit | WB<br>1:1000 | Abcam (ab207494) |
| Anti-GFP, ABfinity Monoclonal | rabbit | 1:50 | Thermo Fisher (G10362) |
| Anti-GFP | mouse | WB<br>1:1000 | Roche (11814460001) |
| Anti-mouse-Atto 594 | goat | 1:1000 | Sigma-Aldrich (76085-1ML-F) |
| Anti-mouse-Atto 647 | goat | 1:1000* | Sigma-Aldrich (50185-1ML-F) |
| Anti-rabbit-Atto 594 | goat | 1:1000* | Sigma-Aldrich (77671-1ML-F) |
| Anti-rat-Atto 594 | goat | 1:1000* | Rockland (ROCK612-155-120S) |
| GFP-Booster_Atto 488 | - | 1:200 | Chromotek (gba488-100) |
| Anti-mouse-800 | donkey | WB<br>1:10000 | Rockland (610-745-002) |
| Anti-rabbit-680 | donkey | WB<br>1:10000 | Rockland (610-744-002) |
| Anti-rat-800 | donkey | WB<br>1:10000 | Rockland (612-145-002) |

**S3 Table. Antibodies used in this study.** List of antibodies with information of species, final dilution and source with order number. Starred (*) are dilutions for confocal IFAs. For STED microscopy concentration was increased to 1:200.
