# Supplementary material for: Malaria parasite centrins can assemble by Ca2+-inducible condensation
Source: PLoS Pathog. 2023 Dec 27;19(12):e1011899. doi: 10.1371/journal.ppat.1011899 (PMC10775985; doi:10.1371/journal.ppat.1011899)
Supplement: Attachment — Submitted filename: 135945_1_rebuttal_2768313_rzlskq.pdf [file ppat.1011899.s031.pdf]

Full Revision

Manuscript number: RC-2023-01967
Corresponding author(s): Julien, Guizetti

1. General Statements [optional]

We would like to thank all reviewers for taking the time to evaluate our manuscript. Many helpful suggestions and discussion points were raised. These comments were instrumental to provide more data that strengthen our conclusion about the relevance of centrin condensation in vivo, expand our findings to other organisms, and improve the manuscript in general. Details are given in the following individual replies.

All line numbers given below refer to the document with the tracked changes.

2. Point-by-point description of the revisions

Reviewer #1 (Evidence, reproducibility and clarity (Required)):

Voss and colleagues show calcium-dependent assembly of Plasmodium falciparum centrins in vitro and in parasites. This assembly is dependent on the EF-hands of centrin and an N-terminal disordered region.

Major concerns:

1. The very definitive title is not wholly supported by the data. This should be qualified by specifying the conditions under which the centrins can accumulate in this way.

We understand this comment by the reviewer. There are multiple dimensions to the potential of centrins to condensate, such as the specific centrin family member, in vivo vs in vitro situation, and media conditions. Naturally it is difficult to represent these various conditions in a concise and compelling title but in line with the suggestion by Reviewer 2 we are changing the title to "Malaria parasite centrins **can** assemble by Ca2+-inducible condensation" to reflect the conditionality of this process.

1. A major concern is whether this behaviour of centrins represents a biologically relevant mechanism in centriolar plaque formation. Is this limited to high overexpression conditions or in vitro high concentrations? Or is it a result of the tagging of the P. falciparum centrins?...

Centrin accumulation at the centriolar plaque and assembly of the centriolar plaque itself must be differentiated. Although compelling we are already very careful in the text about extrapolating our findings about centrin accumulation in cells to centriolar plaque or centrosomal assembly in general. We, however, thank the reviewer for this important comment and now have carried out hexanediol treatment of wild type parasites to test the effect on centrin in a native context. After IFA staining we failed to detect any centrin foci at the centriolar plaques, suggesting that they can be resolved by inhibiting weak hydrophobic interactions that are typical for phase separation (now Fig. 6, lines 283ff).
Concerning the effect of tagging we have generated new data of cells overexpressing an untagged version of PfCen1 in parasites, which still shows formation of ECCAs as revealed by IFA (now Fig. 4H-K, lines 243ff). This significantly alleviates the concern that the observed phenomenon is only a consequence of GFP-tagging. Our in vitro data already showed that native and tagged PfCentrin1 & 3 can undergo condensation.
Concerning the critical concentration of our in vitro assay we find it to be around 10-15 µM without the addition of crowding agents such as PEG (now Fig. S3C, lines 120ff). To our understanding it is challenging to select an in vitro concentration that is adequate to define a threshold for “biological relevance” due to so many additional factors playing a role in vivo. Those factors can also favor a phase separation locally when total saturation concentration is not reached as we now discuss in more detail (lines 440ff). For reference the critical concentration of FUS, which is one of the most studied phase separating proteins in model system, is around 2 µM, but concentrations below 15 µM are well within the range of what is observed for in vitro LLPS. Additionally, it is important to consider that we find Cen1/3 and HsCen2 LLPS is inducible and reversible and that very homologous proteins i.e. Cen2 and 4 serve as an adequate internal control.

… A convincing approach to addressing this issue would be to knock-in a fluorescent tag to the centrin loci. Roques et al. (ref. 12 in this submission) report the GFP tagging of centrin-4 in P. berghei, although they note that centrins-1 to -3 were refractory to tagging in this organism. It is unclear whether Voss et al. attempted this tagging in P. falciparum. This should be clarified and relevant data presented.

We indeed attempted several unsuccessful iterations of tagging Cen1/3 with HA and GFP tag and now explain this in the text more clearly (lines 81ff). We did not attempt tagging Cen2 and 4 as they do not display phase separation in vitro or carry IDRs.

If the tagged molecules used in the biochemical parts of this study are functional, it is challenging to understand why the centrins cannot be tagged in P. falciparum. If the tags render the P. falciparum centrins dysfunctional, the study becomes significantly less useful.

Our data shows that in vitro Cen1-GFP can undergo Ca^{2+}-inducible and reversible LLPS and that GFP-tagged centrins can still localize to the centriolar plaque. Centrin function, however, certainly

goes beyond its ability to condensate and localize. It is easily conceivable that interaction with critical binding partners at the centriolar plaque is inhibited by tagging a protein as small as centrin, which prohibits tagging the endogenous version, while its ability to phase separate remains unaltered. To dynamically study a protein in cells tagging is, however, unavoidable. Even though tagging affects any proteins function to highly variable degree we are still convinced that studying those proteins still provides useful information. Our mutant versions of PfCen1 in vivo shows that non-condensating version display different localization. Importantly, as mentioned above, we now provide images of cells overexpressing an untagged Cen1 version, which still causes ECCA formation (Fig. 5H-K). Ultimately, even though tagged versions might not be fully functional, our observations are compatible with the ability of centrins to condensate in vivo.

3. If a knock-in cannot be achieved, it must be shown that the transgenic expression of tagged Plasmodium centrins does not confound the analysis of centrin behaviour. It is known that these proteins can behave anomalously when overexpressed (Yang et al. 2010, PMID: 20980622; Prosser et al. 2009, PMID: 19139275), at least in other species.

Thank you for this comment. Transgenic expression of proteins can in principle influence their behavior. In the context of this study the overexpression is, however, used intentionally since protein concentration correlates with the phase separation. Here, transgenic overexpression is used as a tool, rather than being a confounding factor, and ECCA formation can be used as quantifiable phenotype. The observation that ECCAs appear significantly earlier the higher they are expressed is in our opinion one of the stronger points of evidence that this result from phase separation in vivo. Yet centrins maintain their centriolar plaque localization and no significant impact on growth is observed. To definitely answer whether phase separation of endogenous centrin is occurring during centriolar plaque accumulation is challenging. These challenges and limitations are now addressed in the significantly extended discussion. As explained above untagged Cen1 also forms ECCAs.

A previous description of centriolar plaque from the authors' lab (Simon et al. 2021, PMID: 34535568) shows an organized structure of an established size. It should be demonstrated whether the structures formed with the GFP tagged centrins show the same dimensions and dynamics as those in wild-type parasites. The extent of the overexpression of the GFP-tagged centrins should also be demonstrated.

We thank the reviewer for this suggestion. We have now added spatial measurements of the centrin signal dimensions at the centriolar plaque of mitotic spindle containing nuclei in PfCen1-GFP overexpressing vs non-induced cell lines. We found that the width of the centrin-signal at the centriolar plaque was unaltered while the height only increased by 11% (Fig. S9). Further, we found no significant growth phenotype in overexpressing parasites, which indicates that the centriolar plaque is functional.

Due to several confounding factors, we were, unfortunately, unable to clearly quantify the extent of overexpression. Most notably the induction of overexpression only works in about 50% of the cells (Fig. S6). The mean intensity after induction further displays quite some variability. Furthermore, the expression kinetics along the IDC of endogenous centrin and our overexpression system that we use as a tool differ. Lastly, our centrin antibodies display crossreactivity (see also Fig. S12) making it impossible to identify how much of the endogenous pool we are labeling in comparison to the GFP- tagged Cen1 protein.

4. It would also be useful to remove the His tag from the recombinantly expressed and purified centrins for the in vitro analyses, particularly if concern remains about the impact of tags on Plasmodium centrin behaviour.

Based on the published in vitro studies on other centrins, we did not anticipate the His-tag to change LLPS properties. Also, Cen1 and 3 and Cen2 and 4 would need to be differentially affected by the tag. We further have experimented with N-terminally tagged 6His-Cen3 protein and found no significant differences in our turbidity assays. Nevertheless, we expressed new versions of the recombinant PfCen1-4 proteins with a TEV cleavage site inserted after the His-tag to purify untagged proteins and found no fundamental differences in our LLPS assay aside some slight variation in the kinetics (Fig. S3E).

5. The discussion is very short and does not consider the findings presented here in the context of the literature, with respect to centrins, Plasmodium MTOC assembly mechanisms, or to general considerations around biological condensates. Andrea Musacchio's recent commentary (ref. 44 in the current submission) advocates caution in ascribing phase separation as an assembly mechanism for organelles in vivo, particularly on the basis of in vitro experiments with high concentrations of homogeneous protein. It is not clear that the concentration dependence of extracentrosomal centrin accumulations (ECCAs) at the onset of schizogony provides sufficient justification of a phase separation model in vivo. The authors' recent description of the involvement of an SFI1-like protein, Slp (Wenz et al. 2023 PMID: 37130129), in the centriolar plaque makes a case for non-homotypic interactions also driving assembly and alternative models for ECCA are not convincingly excluded. The absence of a robust discussion of such considerations is unhelpful to the reader.

We very much thank the reviewer for this suggestion, which helped to significantly improve the manuscript. We have purposefully included the commentary by Andrea Musacchio to highlight a different (possibly the most antipodal) point of view on the role of biomolecular condensation in membraneless organelle formation for the unfamiliar readers that might be just getting to know the field of phase separation. In the absence of word limitations, the reviewer is right to point out the lack of more extensive discussion. We now have significantly extended this section and address the suggested points including the potential role of the novel centriolar plaque protein Slp, which was not published upon submission of our previous version (lines 450ff.)

6. It is also unclear whether the analysis of human centrin is suggested to indicate a phase separation mechanism for centrins in human cells. As this is readily testable, this notion could be considered further. Although its experimental examination may lie outside the theme of this study, one would expect some discussion of the significance of the data presented in the study.

Since it is the first description of phase separation of centrin, it would indeed be interesting to explore the functional relevance in other organisms such as humans. We are considering approaching this in the future. We have, as requested above, significantly extended the discussion and now also include this aspect. Earlier reports have e.g. shown centriole overduplication in human cells upon centrin overexpression.

Minor points

7. There are only three centrins in humans. Centrin 4 is a pseudogene (Gene ID: 729338 on NCBI).

Thank you for detecting this error, which we now corrected (line 60). Centrin 4 seems only to be an expressed gene in mice.

8. Line 175 should say 'temporally', rather than 'temporarily. The Abstract should say 'evolutionarily conserved', rather than 'evolutionary conserved'. 'To condensate' is not ideal as a phrase- 'to form a condensate' would be clearer.

Thank you for those suggestions. The text has been modified accordingly.

Referees cross-commenting

I think the other 2 reviewers have made fair, cogent and constructive points. There is good convergence between the reviewers on the significant issues around the study. These concern in vivo and in vitro effects of tagging and of high concentrations.

Reviewer #1 (Significance (Required)):

The biology of the Plasmodium centriolar plaque is of great interest as an alternative MTOC structure, with obvious additional interest deriving from the role of this organism in malaria. Much remains to be learned about this structure, so the topic of this paper is likely to attract a broad readership. Furthermore, the centrins are a widely-expressed and evolutionarily conserved family of eukaryotic proteins, with multiple roles; a new model for their behaviour, such as is suggested here, would be of interest to many cell biologists.

Review COMMONS

With that in mind, significant additional data should be provided to substantiate the model proposed by the authors.

We appreciate that the reviewer considers our manuscript of interest for a broad audience. We feel that our modifications of the text including a more thorough contextualization and addition of some new experimental data now sufficiently supports our claims.

Reviewer #2 (Evidence, reproducibility and clarity (Required)):

The authors analyzed the properties of the four Centrin proteins of the malaria parasite using a combination of in vitro and in vivo approaches. Their findings indicate that two of the four Plasmodium Centrin proteins, PfCen1 and PfCen3, as well as the human Centrin protein HsCen2, exhibit features of biomolecular condensates. Moreover, analysis of cells overexpressing PfCen1 indicates that such biomolecular condensates become more numerous as cells approach mitosis and are dissolved thereafter.

Major comments

A) A critical point that requires clarification is how the protein concentrations used in the in vitro and in vivo assays (20-200 microM in vitro, and not estimated in vivo) compare to that of the endogenous components. This is important because it may well be that 6His-tagged PfCen1, PfCen3 and HsCen2 can form biomolecular condensates when present in vast excess, but not when present in physiological concentrations. The authors should report the estimated cellular concentration of PfCen1-4, as well as that achieved upon PfCen1-GFP overexpression (on top of endogenous PfCen1), for instance using semi-quantitative immunoblotting analysis. Given this limitation, the authors may also want to temper their title by introducing the word "can" after "centrins".

In the context of phase separation, protein concentration is of course a critical metric. However, in vitro and in vivo concentrations cannot be directly compared as the composition of the surrounding solute has a significant impact on the effective saturation concentration. In vitro we find a saturation concentration for Cen1 of 10-15 µM (Fig. S3C), which is within a range that is frequently found other in vitro studies as listed in the in vitro LLPS data base (PMID: 35025997). We now more explicitly discuss this in the text (lines 422ff). At this point, unfortunately, we have no means of investigating the absolute concentrations of centrin in vivo and to our knowledge no such data is available for apicomplexan. Additionally, one has to keep in mind the presence of other centrin family members in the cell which can interact and co-condensate as well as other

centriolar plaque proteins, like PfSlp, but are difficult to separate through analysis. Further we now discuss several contexts that modify the saturation concentration in vivo (lines 440ff).

As explained above in a response to Reviewer 1, we were not able to produce a satisfactory quantification of the overexpression levels. We are repasting the previous response here:
“Due to several confounding factors we were, unfortunately, unable to clearly quantify the extent of overexpression. Most notably the induction of overexpression only works in about 50% of the cells (Fig. S6). The mean intensity after induction further displays quite some variability. Lastly the expression kinetics along the IDC of endogenous centrin and our overexpression system that we use as a tool differ. Lastly, our centrin antibodies display crossreactivity (see also Fig. S12) making it impossible to identify how much of the endogenous pool we are labeling in comparison to the GFP- tagged Cen1 protein. “

Concerning the title, as explained above, we followed the suggestion and added the word “can”.

B) Movies S1 and S2 (and the related Fig. 1D and 1E) are not the most convincing to support the notion that the observed assemblies are biomolecular condensates, as not much activity is going on during the recordings. Likewise, Movies S3, and even more so Movie S4, as out of focus for a large fraction of the time, making it difficult to assess what happens at the beginning of the process. Moreover, it appears that fusion events, while occurring, are rather rare. The movies should be exchanged for ones that are in focus, and ideally a rough quantification of fusion events as a function of biomolecular condensate size provided.

We thank the reviewer for requesting clarification. Movies S1 and S2 are by no means direct evidence for biomolecular condensation and we do not claim them to be but rather say that they are “…reminiscent of biomolecular condensates…”. We think that this is an appropriate entry into the subsequent analyses. For Movie S1 it is noteworthy that the shape of the accumulation, which can only be resolved by super-resolution microscopy in live cells, is round as would be expected for a liquid condensate in the absence of forces and on these short time scales. Nevertheless, the centriolar plaque must be duplicated which might be the process partly depicted in Movie S2. The observation that centrin can be still change its shape at least suggests that it is not a solid aggregate. In the context of centriolar plaque biology and the technological advance of applying live cell STED in *P. falciparum*, we think these data are still worth reporting.

Concerning Movies S3 and S4 we have carefully selected the focal plane to highlight all the hallmarks of LLPS. Since the protein droplets freely move in 3D throughout the entire imaged liquid volume there is no z-plane that is in focus. Our positioning of the focal plane presents the best compromise between showing round droplet shape, droplet fusion events, and surface wetting. All those observations demonstrate the liquid nature of the condensates. Fusion events are indeed relatively rare, and we do not go beyond this qualitative statement that it can be seen.

C) An important control is missing from Fig. 2, namely assaying PfCen1-4 without the 6His tag, to ensure that the tag does not contribute to the observed behavior (although it can of course not be sufficient as evidenced by the lack of biomolecular condensates for PfCen2 and PfCen4).

Thank you for this suggestion. Since reviewer 1 made a similar comment, I'm reiterating our previous reply here: Generally speaking, and based on the published in vitro studies on other centrins, we didn't anticipate the very small His-tag to change LLPS properties. Also, Cen1 and 3 and Cen2 and 4 would need to be differentially affected by the tag. We further have experimented with N-terminally tagged 6xHis-Cen3 protein and found no significant differences in our turbidity assays. However, we expressed new versions of the recombinant PfCen1-4 proteins with a TEV cleavage site inserted after the His-tag to purify untagged proteins and found no significant differences in our LLPS assay (Fig. S3E).

D) The authors should test whether the assemblies formed by PfCen1 and PfCen3 are sensitive to 1,6-hexanediol treatment, as expected for biomolecular condensates.

This is an interesting and helpful suggestion. We now tested 1,6-hexanediol addition to recombinant PfCen1 and wildtype parasites (now Fig. 6). Interestingly the dissolving effect of hexanediol on PfCen1 in vitro was moderate, which we attribute to the polar component in centrin assembly, which has been documented earlier (Tourbez et al. 2004). In vivo, however, only 5 min of treatment caused a striking dissolution of most centrin foci in wild type parasites, which is compatible with the interpretation that centrin or centriolar plaque assembly could be driven by biomolecular condensation.

E) The fact that HsCen2 also forms biomolecular condensates is very intriguing, but further investigation would be needed to assess the generality of these findings. For instance, the authors could test in vitro also S. cerevisiae Cdc31, the founding member of the Centrin family of proteins to further enhance the impact of their study.

We thank the reviewer for this suggestion. It would of course be exciting to investigate in more detail how widely this biochemical property of some centrins is conserved. To take a first step in that direction, we have recombinantly expressed centrins containing some N-terminal IDRs from *C. reinhardtii, T. brucei* and *S. cerevisiae* to represent organism of significant evolutionary distance. Using our in vitro phase separation assays, we found a very similar behavior to PfCen1 for two centrins while yeast Cdc31, although forming droplets, had a much higher saturation concentration, which could be explained by the significantly lower intrinsic disorder in its sequence (now new Fig. 3).

Minor comments

1) For the experiments reported in Fig. 3D, the same concentrations as those used in Fig. 3A-C (namely 10 microM, and not 30 microM as in Fig. 3D) should be used. Moreover, it would be informative to test whether PfCen2 and PfCen4 as PfCen3 when added to PfCen1.

Unfortunately, this experiment is not feasible since Cen3 does not produce droplets at 10 µM. Hence, in Fig. 3D we aimed to test if Cen1 is incorporated into preformed droplets i.e. whether there is still some interaction between them. We have, however, tested the addition of Cen2 to Cen1 and Cen3 and as expected from the inability PfCen2 to condensate we did not find the same synergistic effect as for Cen1 and 3 together (now Fig. S6). The combination of Cen1/2/3 still enabled co-condensation while addition of Cen4 did not further improve droplet formation. Taken together this strongly suggests that only Cen1 and 3 contribute to the phase separation in vitro (lines 184ff).

2) The authors mention that the effect of Calcium in inducing biomolecular condensates is specific, as Magnesium was not effective (lines 94-95). However, an examination of Fig. S3B indicates that the Magnesium also exhibits some activity, albeit less potent than Calcium. The authors should discuss this point and rectify the wording in the main text.

Thank you for pointing this out. While PfCen1 is not reactive to Magnesium, PfCen3 and HsCen2 do display a small reaction, which we now more clearly mention in the text (lines 118ff). Of note Mg^{2+} and other divalent cation are known to generally promote phase separation.

3) Do the authors think that PfCen2 and PfCent4 localize to the centriolar plaque in vivo using another mechanism that deployed by PfCen1 and PfCent3? It would be good to discuss this point.

This is indeed a point worth discussing. Centrins can of course still interact in the absence of biomolecular condensation and their localization to the centriolar plaque is not dependent on their ability to phase-separate as seen for PfCen2 and 4. We have recently described a novel centriolar plaque protein PfSlp that interacts with centrins and might assist recruitment (Wenz et al. 2023). Cellular condensates are, however, often separated into scaffold proteins, which actually phase separate and client protein which get recruited into those condensates. It is easily conceivable that Cen1 and 3 participate in formation of the biomolecular condensate into which Cen2 and 4 as well as other centriolar plaque proteins might be recruited. Unfortunately, we were not yet able to establish a recruitment hierarchy by e.g. dual-labeling of centrins to test whether PfCen1 and 3 might appear prior to PfCen2 and 4. We now include those aspects in the extended discussion.

4) Given that the EFh-dead mutant exhibits no activity in vitro and fails to localize in vivo, one potential concern is that the protein is misfolded. The authors should conduct a CD spectrum to investigate this.

Thank you for suggesting this relevant control experiment. We have carried out CD spectroscopy of wild type and EFh-dead PfCen1 and find no difference in secondary structure distribution. We now added these data to the supplemental information (now Fig. S14).

5) It is not entirely clear from the main text in lines 103-104, as well as from the legend, what Fig. S3B shows. When was EDTA added in this case?

Thank you for requesting clarification. We will assume the reviewer is referring to Fig S4B. We wanted to show that contrary to PfCen3 that PfCen1 droplets can still be resolved after an elongated period of incubation with calcium but forgot to mark the timepoint of EDTA addition at 180 min in the graph. We have now corrected this and further reworded the sentence for more clarity (lines 132ff).

6) Fig. S7: the correlation between PfCen1-GFP expression levels and ECCA appearance is modest at best. What statistical test was applied? This should be spelled out. Moreover, the authors should combine the two data sets, as this will provide further statistical power to assess whether a correlation is truly present.

Indeed, the correlation is modest but statistically significant, which is why we decided to place this data in the supplemental information. The used statistical test was an F-test provided by Prism, which compares two competing regression models, which we now mention in the legend. Combining the two data sets is unfortunately not possible since they arise from two independent sets of measurements where different imaging settings had to be used to adjust for the very different fluorescent protein levels in both lines after induction.

7) The authors may want to discuss how their findings can be reconciled with the notion that Centrin assemble into a helical polymer on the inside of the centriole (doi: 10.1126/sciadv.aaz4137).

This is an interesting point. Although centrin does localize to the inside of the centriole (https://doi.org/10.15252/embj.2022112107), more precisely one pool at the distal part and one pool at the core, there is no evidence that it is itself part of the helical inner scaffold described by the authors even though it might localize in close proximity to it. Further, there are several examples where polymers such as microtubules act as seeding point for biomolecular condensates or the other way around, and our work suggest this could be a potential working model for centrins. We have discussed our results extensively with the two corresponding authors of the aforementioned study (i.e. Virginie Hamel and Paul Guichard) and agreed that our data are not conflicting. Nevertheless, we include the inner centriole localization and potential association with polymer structures of centrin in our extended discussion.

9) Likewise, the authors may want to speculate regarding what their findings signify for the role of Centrin proteins in detection of nucleotide excision repair (doi: 10.1083/jcb.201012093).

We appreciate the comment by the reviewer. Centrins seem to have many different potential roles that remain to be clarified. While we are excited about this, we think it is too early to speculate about the impact of centrin condensation on less well studied aspects of centrins such as nucleotide excision repair. We, however, now cite this study in the discussion to highlight the functional diversity of centrins.

Small things

- Fig. 1A: change color for microtubules as red on red is difficult to discern.

Throughout our publications we use this shade of magenta to label microtubules in schematics and have therefore opted to use a slightly brighter shade of red for the RBCs instead to improve visibility.

- Fig. 1C: the indicated boxes in the top row do not seem to correspond exactly to the insets shown in the bottom row.

We have verified the position of the boxes and found them to be accurate. Possibly the different imaging modality used for both panels (confocal vs STED) creates this impression.

- line 266: typo, promotor > promoter.

Has been corrected.

- line 360: a reference should be provided for the GFP-booster, including the concentration at which it was used.

Has been added.

- line 363: "an" missing before "HC".

Has been corrected.

- line 428: it would be best to deposit the macros on Github or an analogous repository.

Macros have been deposited on https://github.com/SeverinaKlaus/ImageJ-Macros (line 737)

- line 461: "to the" is duplicated.

Has been corrected.

- Fig. S5A: maybe draw the lines in red (as red in Fig. S5B correspond to the proteins that do not have IDRs).

Since we cannot easily change the line colors of the IDR graphs, we have inverted the font color for Fig. S5B instead.

- Movie S7, legend: left frames shows PfCen1-GFP, not microtubules as currently stated.

Has been corrected.

Reviewer #2 (Significance (Required)):

This is a provocative study that extends initial observations regarding self-assembly properties of Centrin proteins, and posits that some members of this evolutionarily conserved family can form biomolecular condensates. After the above outstanding issues have been properly addressed, these data could have important implications for understanding Centrin function in centriole biology and DNA repair. Therefore, these findings will be of interest to a cell biology audience.

Field of expertise: cell biology.

Reviewer #3 (Evidence, reproducibility and clarity (Required)):

Summary:

The authors have provided a comprehensive characterisation of centrin proteins in Plasmodium falciparum. Through expression of episomal GFP-tagged centrin for in vitro, they were able to observe co-localisation of centrin with centriolar plaques during the replicative stage of the parasite. They also utilised live cell STED microscopy to track dynamic changes in centrin morphology. They have also demonstrated calcium-dependent phase separation dynamics in bacterially-expressed P. falciparum centrin and human centrin 2. The formation of liquid-liquid phase separation in PfCen1, 3 and HsCen2 tied well with IUPred3 predictions of intrinsically disordered regions in these proteins. Using an inducible DiCre overexpression system with two promoters of varying strengths, the authors have shown accumulation of centrin1 outside of centrosomes and premature appearance of centriolar plaques. Finally, changes on the centrin1

protein, i.e., N-terminal deletion, and mutations in calcium binding sites in the EFh domains, have shown a reduction in the formation of ECCAs during overexpression and inability to form LLPS in vitro, respectively.

Major comments:

1. Given that parasites cannot tolerate endogenous C-terminal tagging of some centrins (but not all, as PbCen4 was successfully tagged), has N-terminal tagging been attempted either by the authors or in previous publications? Note that this is not a request for further experimentation; rather, maybe this can be noted in the manuscript; and line 62 can be rephrased for transparency.

We have not attempted N-terminal tagging ourselves but through personal communication with Rita Tewari we were informed that neither N- nor C-terminal tagging for PbCen1-3 was successful in the context of the study published by Roques et al 2018. We have only unsuccessfully attempted C-terminal tagging in several iterations. Due to importance of N-terminus for interaction and function in other organisms it is plausible that N-terminal tagging is even more unlikely to work. Since we have not exhaustively attempted every tagging strategy on every centrin we, as suggested, rephrased the text accordingly (lines 81ff).

2. Is there a possibility that by adding a C-terminal tag, centrin may lose a specific function or cause change in the physicochemical properties of the protein (thus making C-terminal tagging lethal)? Was His tag removal attempted so the native protein can be used in the LLPS experiments? IUPred3 analysis showed potential IDR at the C-terminal end of PfCen4. Could the C-terminal tag have caused the protein to not form droplets in the presence of Ca2+?

As we could show for PfCen1-GFP, the tag did not impair its ability to undergo LLPS which is at least partly mediated by the N-terminus, and that it could still properly localizes to the centriolar plaque. The fact that some endogenous centrins cannot be tagged suggest that there is a functional relevance to the C-terminus that could e.g. be an interaction with other essential centriolar plaque components. As suggested in a reply to Reviewer 1, we consider a substantial and centrin-specific effect of the small His-tag on phase separation unlikely. To be sure, we have repeated our turbidity assays with tag-free versions of PfCen1-4 and found no change in phase separation properties (now Fig. S3E).

3. It has been shown by the authors that different tagged centrins co-condense which may support the localisation data (Figure 1C). However, is there a way to show that the episomally- and endogenously-expressed centrin co-localise with each other (e.g., confocal microscopy with anti-centrin vs anti-gfp in PfCen-GFP lines, that is if the authors have access to anti-centrin antibodies)? Has endogenous centrin been demonstrated to form ECCAs (in previous publications or by the authors)?

These are important questions by the reviewer. Due to the high sequence homology centrin antibodies, even if raised against a specific centrin (such as PfCen3 in this study), will likely cross-react with other centrins. So far, we have not been able to produce a staining were the anti-GFP-positive foci are devoid of anti-centrin3 staining, which limits the interpretation of these data. The outer centriolar plaque compartment containing centrin is, however, well defined by now and the localization pattern of endogenous centrin and Centrin1 and 4-GFP seems identical. In a more recent study from our lab Cen1-GFP IP has identified other endogenous centrins as interaction partners (Wenz et al 2023), like the Roques et al. 2018 study did for PbCen4-GFP indicating that the tag does not abolish interaction between centrins. So far, we have never detected any ECCAs, nor have we identified any similar structure in the literature. This suggest that this is indeed a consequence of excessive centrin concentration. Importantly we now have added data from a new parasite line overexpressing untagged PfCen1 using the T2A skip peptide (pFIO+_GFP-T2A-Cen1) which displays ECCAs upon induction, showing that this effect is not a mere consequence of tagging (now Fig. 5H-K).

Minor comments:

1. How were the times (post addition of Ca2+) presented in Figure 2A determined?

We noted down the time of calcium addition and cross-referenced it with the timestamps available in the metadata of the movie files (e.g. file creation timepoint marks the start of the movie). We now mention this in the legend.

2. Line 126: Figure 1B should be Figure 1C
3. Line 145: Figure 1C-D should be Figure 1D-E
4. Line 151: Figure 3A should be Figure 4A

Thank you for spotting these mistakes, which now have been corrected.

5. Line 152: Suggest rephrasing "placing the gene of interest in front of the promoter" to "placing the gene of interest immediately downstream of the promoter" or something similar

Thank you for this good suggestion.

6. Any growth phenotype changes observed in the overexpressors?

The parasite lines seem to silence the Cen1-4-GFP expression plasmids readily, which suggest that there might be a growth disadvantage. However, repeated attempts to quantify a growth phenotype were unsuccessful due to high variability in the data, which might be partly connected to the fact that the fraction of GFP positive cells after induction can vary between lines and replicas.

7. How often are ECCAs observed in pARL strains, or are they not observed at all? This might be good to mention.

ECCAs in the pArl strains have been observed on very limited instances but are too rare to be quantified. We now mention this in the text (lines 217ff).

8. Line 192 and Figure S8: n {less than or equal to} 33 (either a typographical error and should have been {greater than or equal to}, otherwise, it may be expressed as a range)

It was indeed a typographical error that was now corrected.

9. Line 258: Methods on the generation of FIO/FIO+ was a bit difficult to understand. Maybe a simple plasmid schematic with the restriction sites (at least for the original plasmid) in the supplementary may help clarify this.

Cloning strategy has been expanded with additional information for clarity.

10. Line 295: include abbreviation of cRPMI here rather than in Line 303

Has been corrected.

11. Line 322: typographical error on WR99210 working concentration?

Has been corrected.

12. Line 372: Last sentence on area and raw integrated density measurement is unclear.

We have reformulated the sentence for more clarity.

13. Line 461: typographical error in last sentence

Has been corrected.

14. Line 532: Figure 4E should be Figure 4F

Has been corrected.

Full Revision

Reviewer #3 (Significance (Required)):

DNA replication is vital to the survival of malaria parasites. A deeper understanding on their unusual form of replication may be exploited to find drug targets uniquely directed to the parasite. Biological insights from this work can also provide a jump-off point for unravelling unusual replication in other organisms. Data on the physicochemical analysis of centrin is not just of great interest for those in the field of parasitology, but also for those in the much wider fields of biology, physics and chemistry. Techniques presented in this work (e.g., DiCre overexpression with different promoters) can definitely be utilised for the elucidation of protein function within and outside the field of parasitology.

My field of expertise is in Plasmodium spp., particularly in parasite replication, molecular and cellular biology, and epigenetics.

We thank the reviewer for the appreciation of our work in terms of insight and technology development.
