# Supplementary material for: Malaria parasite centrins can assemble by Ca2+-inducible condensation
Source: PLoS Pathog. 2023 Dec 27;19(12):e1011899. doi: 10.1371/journal.ppat.1011899 (PMC10775985; doi:10.1371/journal.ppat.1011899)
Supplement: Attachment — Submitted filename: Response to reviewers.pdf [file ppat.1011899.s032.pdf]

<u>Response to reviewer comments</u>

Part I – Summary

Reviewer #1: The revision of this submission has greatly improved it; the issues raised in my initial review have been satisfactorily addressed and I consider that the data in the paper now support the major conclusions drawn in the study. The expanded discussion positions this work in the context of the literature and will allow readers to consider the ramifications of the authors' findings in several different directions. I think this paper makes a valuable and interesting contribution to centrosome biology.

Reviewer #2: Plasmodium falciparum survival is very much dependent on its ability to efficiently replicate and one important component of the nuclear replication machinery is centrin. Understanding this can help identify novel drug targets against a parasite that is well known to have developed resistance to almost all anti-malarials. The study has extensively characterised centrins in P. falciparum. The authors have described centrin in normally replicating parasites as well as in the case of protein overexpression in vivo, both instances providing important insights on centrin. Although episomally expressed tagged proteins were used due to the difficulty of tagging the endogenous protein, necessary controls were done, further supporting the data being presented here. The study also covered in vitro characterisation of centrin, demonstrating LLPS in the presence of calcium (but not magnesium), and reversed in the presence of EDTA. They have also conducted similar experiments involving centrins from other evolutionarily distant eukaryotes with predicted intrinsic disordered regions. This therefore will be of great interest to a wider audience, and not just in the malaria community or parasitology. Perturbations on the centrin protein (i.e., removal of the N-terminal intrinsic disordered regions, EFh mutation) demonstrated the importance of these protein regions in liquid-liquid phase separation. The authors have provided circular dichroism spectroscopy to ensure that the mutations do not affect the protein's secondary structure.

Reviewer #3: The authors conducted several experiments and analyses to address the comments raised by the three reviewers of the initial submission. The manuscript is clearly improved as a result. In my opinion, however, a few outstanding issues remain to be clarified before publication could be endorsed, as detailed hereafter.

We are very grateful for the constructive feedback by all reviewers, which made the significant improvements possible. We further appreciate the positive comments on our work and will address the remaining concerns in the following.

Part II – Major Issues: Key Experiments Required for Acceptance

Reviewer #1: (No Response)

Reviewer #2: No major issues

Reviewer #3: Major points

The major remaining outstanding issue pertains to the relevance of these observations to the behavior of PfCen1 and PfCen3 in vivo, an issue highlighted also by Reviewer 1 and to some extent by Reviewer 3 in their assessment of the initial submission. Obviously, the best way to address this would be to endogenously tag PfCen1 or PfCen3, which the authors argue they cannot do (although they did not attempt N-terminal tagging). In the absence of endogenous tagging, could the authors provide cells with plasmids where PfCen1-GFP or PfCen3-GFP are driven by their respective endogenous promoters? Without such experiments, one is left wondering whether ECCAs might simply result from proteins being overexpressed at non-physiological levels. The authors argue that overexpression experiments are by design (" the overexpression is, however, used intentionally since protein concentration correlates with the phase separation"), but given the very weak correlation in question (see Fig. S8), perhaps overexpression in all cases is well above the levels of endogenous components, which would then bring little understanding to how these proteins function in regular cell physiology.

We thank the reviewer for requesting clarification. We indeed would have liked to test endogenous tagging of the essential centrins if it could have been realized. Extensive N- and C-terminal tagging of Cen1-4 with GFP, mCherry, or HA-tag has been attempted in the related parasite *P. berghei* (Roques et al. 2019 and personal communication with Rita Tewari). Due to the very high sequence identity between *P. berghei* and *P. falciparum* centrins (Cen1: 96%, Cen2: 97%, Cen3: 94.97%, Cen4: 68.83%) we did not try N-terminal tagging after our unsuccessful C-terminal tagging attempts. We appreciate the reviewer's suggestion of using constructs driving centrin expression under their endogenous promoter. While it is not feasible to develop and test these new constructs within the time given for this revision, we are considering including such constructs in future analyses. Also, as suggested in the minor comments, we now expand on the attempted tagging strategies in the text (lines 574ff).

Importantly, however, we seem to not have communicated clearly enough the purpose of overexpression via the pFIO plasmid as well as the nature of ECCAs in the context of this study. ECCAs are indeed a consequence of overexpression and are not observed in wild type cells or only very rarely in strains expressing PfCen1/3 from the pARL expression plasmid, which has a weaker promoter (Fig. 1C). ECCAs are likely not desirable for cells regarding their propensity to "rip-off" from the centriolar plaque. pFIO-induced overexpression was specifically used as a tool to modulate one of the defining aspects of phase separation in the cell, i.e., protein concentration. Indeed, we found earlier ECCA formation and more ECCAs in cells with higher PfCen1-GFP overexpression. In addition, mutating PfCen1 (either by removing its N-terminus

or by impairing Ca2+ binding via the EFh domains), led to a loss of ECCA formation despite overexpression. This is compatible with centrins being able to also undergo phase separation in vivo. Whether endogenous centrins undergo phase separation at native concentrations cannot be fully answered at this point. Our data suggests that in principle, they have the ability to do so. If endogenous tagging of centrin was possible, it would be difficult to exclude with certainty that tagging itself would alter protein behavior as an intrinsic limitation of this type of research. Here, our experiments with mutant centrins and hexanediol treatment provide complementary evidence. We now have clarified the text regarding overexpression experiments and the nature of ECCAs (lines 209ff, 419ff).

Concerning Fig. S8 (now Fig. S9), the centrin expression level variability within a single parasite strain expressing either pFIO or pFIO+, shown in two separate graphs, is indeed not very high. Confounded by general variability between individual cells, this resulted in a weak but still significant correlation between ECCA amount and PfCen1-GFP levels. We have placed these data only in the supplements because it is more relevant to compare the clear phenotypes caused by high difference in expression levels, i.e., between pFIO and pFIO+ shown in Fig. 5. We now explain this more explicitly in the text (lines 210ff).

Another way to mitigate this critical concern would be to show that the level of overexpressed PfCen1-GFP or PfCen3-GFP approximates the levels of the corresponding endogenous proteins. Despite the arguments presented by the authors, it is not clear why they could not estimate the extent of overexpression using PfCen1 antibodies, despite the cross-reactivity towards PfCen3. For instance, they could conduct semi-quantitative immunoblot analysis, taking into account that only ~50% of cells are GFP-positive (see Fig. S7), or else perform immunofluorescence analysis. Despite the cross-reactivity with PfCen3, this should allow them to place a lower bound on the extent of overexpression. If variability of expression levels between cells is an issue, these analyses could be conducted following FACS sorting. Moreover, the authors could also envisage conducting semi-quantitative proteomic analysis, should they want to ascertain this question beyond doubt.

This is of course a critical point and in the revised manuscript, we now clearly state that we were not aiming to replicate physiological levels of centrin with our system, but specifically aim to increase centrin concentration to visualize whether phase separation like behavior in vivo is possible in principle. As suggested and to more clearly show the levels of overexpression caused by the pFIO+ system, we now provide semi-quantitative immunoblot analysis and estimate the degree of overexpression at about 18:1 above physiological PfCen1 levels, while considering induction in ~50% of cells. We have added the data as new Fig S8 (see also below) and described the findings in the text (lines 202ff). This serves to further clarify to the reader the use case of the pFIO system in this study and prevent confusion about the interpretation of the data.

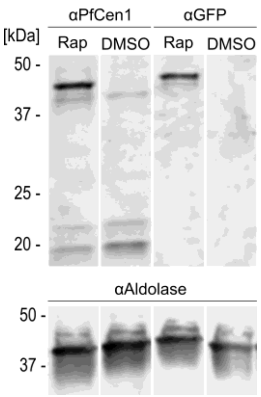

S8 Fig. Overexpression levels of pFIO+_PfCen1-GFP. *Western blot analysis of purified 3D7-DiCre_pFIO+_PfCen1-GFP late-stage parasites treated with rapamycin or DMSO (control) in the previous cycle. The signal of endogenous centrin (19.6 kDa, 93 AU) and PfCen1-GFP (46.5 kDa, 717 AU) stained with rat anti-PfCen1 was quantified. Corrected for PfCen1-GFP being only present in a subset of the population (43%, n = 662, from three replicas), as quantified via microscopy, an average (induced) cell has an approximate PfCen1:PfCen1-GFP ratio of 1:18. Staining with anti-PfAldolase as employed as a loading control. Anti-GFP staining indicated no cleavage of PfCen1-GFP, with no expression being detected in the DMSO treated populations with either staining. An additional band at ~21 kDa in the anti-PfCen1 staining is likely the result of cross-reactivity with PfCen3 (20.9 kDa).*

One argument the authors use to argue that overexpression does not impact cell physiology is the absence of significant growth phenotype (see response to Reviewer 1: "Further, we found no significant growth phenotype in overexpressing parasites, which indicates that the centriolar plaque is functional."). However, in response to Reviewer 3, they write that the "parasite lines seem to silence the Cen1-4-GFP expression plasmids readily, which suggests that there might be a growth disadvantage", mentioning also that they could to assess this with certainty. Taken together, these comments leave me confused regarding whether there is a growth disadvantage or not. Also, if variability in GFP expression levels is an issue to address this with certainty, the authors could sort cells using FACS and analyze the growth of cells as a function of GFP levels. Overall, this point is important to clarify because a growth defect would be indicative of overexpression interfering with cell physiology.

We thank the reviewer for raising this point and providing us with the opportunity to resolve this apparent discrepancy. These statements resulted from two different observations: The "silencing effect" (i.e., low percentage of fluorescent cells) observed for pARL-PfCen1-4-GFP was assessed several weeks after transfection once parasites reappeared in culture and were ready for analysis. There we saw a significant proportion of them, despite selection drug resistance, having reduced or silenced PfCen-GFP expression. In another exploratory experiment, the absence of an observable growth defect was assessed in the next cycles right after induction of pFIO+-PfCen1-GFP carrying parasites and showed no clear growth difference compared to the uninduced population. Hence, overexpressing PfCen may have an adverse effect on parasite fitness, which could select for non- (or low-) expressing parasites over longer time scales. Since we did not detect a growth phenotype after induced overexpression, we think it is unlikely that overexpression of PfCen causes secondary phenotypes, which could affect our experimental analysis. Still, these are for the moment anecdotal observations, which need a more detailed follow up in a future study.

In response to my comment B), the authors argue that Movies S1 and S2 are not meant to demonstrate the presence of biomolecular condensates,…

We purposely avoid the claim that Movie S1 and S2 can only be explained by biomolecular condensation. They, however, display dynamic rearrangement of PfCen1-Halo compatible with biomolecular condensation and may show interesting transition points in the centriolar plaque duplication process through live cell STED microscopy.

…and that the focal planes in Movies S3 and S4 have been selected to highlight the hallmarks of LLPS. What I see in Movie S3 is that the few droplet fusion events (e.g. at 9 seconds into the movie) are, in fact, out of focus. From their reply ("… throughout the entire imaged liquid volume…"), I assume that the authors acquired Z stacks, so that they should choose better focal planes to illustrate such fusion events. As for Movie S4, it appears rather to show that droplets do NOT fuse, as many that appear in physical proximity do not coalesce (again, the fact that the focal plane is usually not the best makes it difficult to say for sure), despite PfCen3 being present at 200 microM. This would argue against LLPS occurring in this case. Could it be that PfCen1 and PfCen3 differ in this respect? Also, a key expectation of LLPS is that droplet size should increase over time. Whereas this might be happening with PfCen1 (although it is difficult to say for sure in the absence of quantification), this does not appear to be the case for PfCen3. The important points must be clarified by further analyses.

Of course, we were keen on capturing fusions, wetting etc. in the optimal focal plane, but we were not able to acquire z-stacks. These were omitted in favor of high acquisition rates to capture the rapid dynamics of centrin droplets. This also meant that we could not apply an autofocus to compensate for an unfortunate gradual focus drift. While this resulted in a reduced chance to capture a fusion event that is precisely in focus, several fusion events of PfCen1 and 3 droplets are nonetheless recognizable (see also Fig. 2A, inserts). In addition, surface wetting, which is another specific hallmark of liquids, can also be observed.

PfCen3, indeed, behaves differently than PfCen1 in vitro. This is best explained by the well described process of maturation where liquid-liquid phase condensates transition into a more solid- or gel-like state (see also lines 100ff). PfCen3 is more prone to maturation as the time-dependent lack of reversibility by EDTA suggests (Fig. 2B and S4). The impairment of droplet-droplet fusion and size increase in later stages, which the reviewer notes, is likely a direct consequence of the maturation process. Nevertheless, the fact that PfCen3 droplets initially form via LLPS is supported by our data (Movie S3, Fig. 2A, lines 96ff). In addition, we have acquired a movie of PfCen3 droplets at 32°C, where maturation is less pronounced than at 37°C, which shows more extensive fusion and wetting (new S7 Movie, lines 121ff). We have further expanded our explanation concerning differential PfCen3 behavior (lines 100ff, 119ff).

Part III – Minor Issues: Editorial and Data Presentation Modifications

Please use this section for editorial suggestions as well as relatively minor modifications of existing data that would enhance clarity.

Reviewer #1: I have a couple of minor points to tidy up before final publication.

1. The reference list should be reviewed for typos, omissions, repetitions etc. There are a lot of mistakes- one presumes this is due to the referencing software, but it is important to get this right in the final version.

We thank the reviewer for pointing out our errors in this important matter. We have now carefully monitored and corrected the references.

2. Some minor typos:

Line 156 'evolutionarily'
Line 188 (and 435, 446, 447, 456): 'to condensate' is not a verb. It should be replaced by 'to form a condensate', 'to demonstrate condensation', 'to undergo condensation' etc.
Line 217 'reevaluating our'
Line 244 'pFIO where GFP'
Line 257 'not dependent on'
Line 469 'attributed'
Line 880 'n =' (I presume).

We are grateful to the reviewer for spotting these typos, which we should have caught ourselves, and have now corrected.

Reviewer #2:

We want to thank Reviewer 2 for careful monitoring of our manuscript and highlighting the following required corrections.

1. Line 217: "When reevaluating our earlier data from the weaker expressing pArl-Cen1/3-GFP parasite lines…"
Remove "of"

Has been corrected.

2. Line 243: "To exclude that ECCAs only form due to GFP-tagging we designed a version of pFIO were GFP and PfCen1 were separated by a T2A skip peptide (Fig 5H)."
Should have a "+" after pFIO

Has been corrected.

3. Figure 5H
Promoter in the figure should only show hsp86 (instead of hsp 70 or 86) to represent pFIO+

Has been corrected.

4. Line 293: "Taken together these observations argue for condensation being part of the mechanism of centrin assembly at the centriolar plaque."
Remove "a"

Has been corrected.

5. Line 581, 600, 638, 681, 741 …Update referencing format

Has been corrected.

6. Line 674: " IFA staining against…"
Remove "A"

Has been corrected.

7. Line 690: Live cells
Change "Life" to "Live"

Has been corrected.

8. Line 693: "264x264 pixel images were acquired L HC PL APO CS2 63x/1.4 N.A. oil immersion objective was employed with GaAsP detectors, a pinhole of 0.6 airy units, a pixel size of 35.1 nm and z-stack of 7.28 µm at 130 nm intervals."
Kindly rephrase

Has been corrected.

9. Standardise use of "6His" vs "6his" vs "6xHis"

Has been standardized throughout the manuscript to 6His

10. Figure S3: missing caption/legend for S3E

Has been corrected.

Reviewer #3: Other points

In an important experiment, the authors tested whether overexpression of untagged PfCen1 also yields ECCAs, finding that this is the case in ~50% of cells in the control (DMSO) condition, and ~80% of cells upon rapamycin addition. Why do ~50% of control cells already have ECCAs? Is this is due to leaky expression? If so, why is this not the case with the constructs inducing PfCent1-GFP expression?

We thank the reviewer for bringing our attention to this issue. Our labelling of the new Fig. 5J-K and the description in the text was indeed misleading. Uninduced control cells (just as wild type) do not display ECCAs. We further have never observed any leakiness in the pFIO+ system prior to induction (Figs S7B, new S8). Since we were analyzing untagged centrin, we had to use a polyclonal antibody we raised against PfCen1. Unfortunately, this antibody despite detecting a specific band and centriolar plaque signal also caused some background staining (Figs 5I, S12). These background staining dots are not readily distinguishable from actual ECCAs caused by PfCen1 overexpression. For the quantification, we applied a threshold based on the signal seen in centriolar plaques to mitigate the effect of unspecific signals from the antibody. We inadvertently designated all signals that surpassed the threshold as ECCAs (line 263, Fig. 5JK), even though in the case of the uninduced control those were likely non-centrosomal background staining. In case of induced cells, we were quantifying non-centrosomal background staining plus ECCAs. To clarify this, we have re-labeled the figures, using the term "non-centrosomal foci" and re-formulated the figure legend and text accordingly (lines 260ff).

In response to comment 7 of my initial review, the authors write that their data do not conflict with the previously reported notion that Centrin is part of a helical inner scaffold, and mention that they have discussed this matter with the corresponding authors of the study in question, who are said to be in agreement with them. This is surprising in light of what is stated in that manuscript (e.g. the abstract states "POC5, POC1B, FAM161A, and Centrin-2 localize to the scaffold structure along the inner wall of the centriole MTTs.").

Indeed, these points of view appear contradicting. However, at this moment we do not know enough to confirm a generalized model for centrin organization. It is however clear that can centrins display a range of localizations patterns throughout eukaryotes (including centrioles, basal bodies, nucleus, contractile fibers, nuclear pores, etc.) and our study also shows that not all centrins can undergo LLPS. This leaves the option for a variety of structural organization. Whether centrins can form macromolecular structures or to which degree they might be an integral building block of a structured complex is unclear at this point (see also lines 364ff). We can only reiterate that during our discussion of this project, Dr. Hamel and Dr. Guichard did not note a fundamental discrepancy with their data. In LeGuennec et al. the authors indeed state that "Centrin-2 localizes to the scaffold" rather than Centrin-2 "being a component" of the inner scaffold. The idea that centrin is an element of the highly structured inner scaffold is speculative at this point. Their data further suggests that Centrin is the inner-most protein of the centriole and could also bind to the surface of the scaffold. We think that this does not preclude

phase separation properties of centrins as e.g. proteins that form condensates on the cytoskeleton, such as the protein tau on microtubules, while not being integral to the structure itself have already been described. In their most recent work, the Hamel and Guichard groups also describe centriole associated centrin pools that are not colocalized with the inner centriole scaffold (Laporte et al. 2022).

The authors write (lines 83-84) that “our experience confirms that tagging of the C-terminus of PfCen1 and 3 is not achievable even using small tags”, they should spell out in some more detail what they tried (e.g. in the Materials and methods section). Otherwise this information is of limited use to the community.

We thank the reviewer for this suggestion and apologize for not including this information earlier. We now mention this in the methods (lines 825ff).

The authors write (lines 121-123) that “the critical saturation concentration for PfCen1-GFP is well within the range of what can be found for in vitro phase separation of other proteins”, referring to the Li et al, 2020 manuscript. However, an exploration of the LLPSDB data base reported in that manuscript does not seem to provide such values. This should be clarified, perhaps by providing a few exemplary values for readers to appreciate this point.

To access the values for the used protein concentrations one must download the data provided as excel files in the download section of the website. We have added the url for the LLPSDB in the manuscript for easier access (line 111). At this point, the database, which is constantly updated, contains well over 2000 experimental datasets for natural proteins ranging in concentration from 1 to 1000 µM. As many different factors affect LLPS, we are not able to make a sound argument for the selection of a small subset of examples, but rather offer the curious reader the opportunity to form their own opinion on this complex matter.

The authors write (lines 286-287) that the fact that the dissolving effect of hexanediol of PfCen1 in vitro was moderate could be attributed to the polar components of the protein. Is this this also the case for PfCen3? Regardless, how do the authors imagine that the postulated impact of such polar components is shielded in vivo?

For self-interaction of centrin in vitro, a significant polar component has been demonstrated (Tourbez et al., 2004; Conter et al., 2021) while hexanediol will predominantly affect weak interactions. Currently, we have not tested the effect of hexanediol on PfCen3 as its maturation provides a confounding factor. How the more complex nature of the cytoplasm could impact the centrin interaction forces in a way that hydrophobic interactions are favored is unclear. We have added a short statement that addresses this (lines 299ff).

The authors write (lines 292-293) that “We further detected a disorganization of the mitotic spindles, which seemed longer and less dense.” This is not apparent from the figure panels. The authors should highlight these feature and, to the extent possible, quantify them.

In our view the morphological changes to spindle morphology a very noticeable in that mitotic spindles are much longer and less dense while classic hemispindle morphologies are barely detectable anymore. We have undertaken a quantification and found that while average mitotic spindles in wild type cells range around 550 nm in length, we frequently detected microtubule bundles largely exceeding 1 µm in 1,6-hexanediol treated cells, i.e. 28 out of 31 compared to 1 out of 26 in untreated control cells. However, since we consider this a peripheral observation, which it is not relevant to the conclusions and is still under investigation we choose to remove the statement.

Line 358-359: the authors mention that Centrins are conserved in all eukaryotes analyzed. To my knowledge, there are no Centrins in nematodes such as C. elegans, nor in insects such as Drosophila. The writing should be rectified to reflect this fact.

This is an important point. Possibly for historic reasons, in nematodes and insects (and frequently algae) a different term is used and centrin is called caltractin. Indeed, caltractin is the inaugural name (Huang et al. JCB 1988) but centrins and caltractins are orthologous. Similarly, in fungi the literature largely refers to Cdc31 instead of centrin.

In the Material and methods (lines 817-821), the authors report how PfCen1 antibodies were raised, but not how they were affinity-purified. This information must be provided, also for better understanding why these antibodies cross-reacts with PfCen3 but not with the other two Centrin proteins.

We now more clearly stated in the materials and methods (lines 822ff) how this antibody was produced and state that the polyclonal rat anti-PfCen1 antibody has not been affinity purified. Instead, serum was used for all experiments, as now indicated. All four centrins have a high degree of homology and we would reason that it is probably difficult to predict whether and to which degree an individual polyclonal antibody would cross react with one or more centrins. Although PfCen1 has the highest sequence identity with PfCen3 (PfCen1 v PfCen2: 51%, PfCen1 v PfCen3: 65%, PfCen1 v PfCen4: 39%).
